# Long‐Term Changes in Planktonic Ciliate Beta Diversity Under Contrasting Hydrological Regulation in A River–Floodplain System

**DOI:** 10.1111/1462-2920.70421

**Published:** 2026-09-16

**Authors:** Yasmin Rodrigues de Souza, Matheus Henrique de Oliveira de Matos, Florencia M. Monti Areco, Melissa Progênio, Bianca Ramos de Meira, Andressa Crystine Souza‐Silva, Felipe Rafael de Oliveira, Luiz Felipe Machado Velho

**Affiliations:** ^1^ Universidade Estadual de Maringá Programa de Pós‐Graduação Em Ecologia de Ambientes Aquáticos Continentais‐PEA, Núcleo de Pesquisas Em Limnologia, Ictiologia e Aquicultura Maringá Paraná Brazil; ^2^ Centro de Ecología Aplicada del Litoral (CECOAL‐CONICET) Corrientes Argentina; ^3^ Universidade do Estado do Mato Grosso (UNEMAT) Cáceres Mato Grosso Brazil

## Abstract

This study assessed temporal beta (β) diversity of planktonic ciliates over 14 years (2010–2024) in the Upper Paraná River floodplain, comparing a dam‐impacted system (EID) and a non‐impacted, protected system (ENID). We tested predictions regarding temporal variation in richness, abundance, composition, and community structure. Both systems exhibited declines in richness and density, more pronounced in EID. Temporal β diversity (TBI) was higher in the impacted system, indicating stronger compositional turnover over time. The local contribution to β diversity (LCBD) values were also elevated mainly in EID, suggesting greater local ecological uniqueness in impacted environments—contrary to expectations for protected areas. The species contribution to β diversity (SCBD) results showed that common, widespread taxa contributed more to β diversity than rare species, emphasising the role of dominant taxa in structuring temporal variation. Variation partitioning indicated that abiotic factors were the primary drivers of community dynamics in both systems, while temporal effects were secondary and similar between them. Overall, the findings demonstrate that conservation status alone does not ensure ecological integrity in floodplain systems. Instead, maintaining natural hydrological regimes—especially flood pulse dynamics—is critical, as these processes regulate connectivity, dispersal, and community renewal, ultimately sustaining biodiversity even in nominally protected environments.

## Graphical abstract

Hydrological regulation by dams reshaped ciliate biodiversity over 14 years, increasing temporal compositional change and ecological uniqueness while accelerating declines in richness and density. These results highlight flood‐pulse dynamics as a fundamental mechanism sustaining biodiversity and ecological integrity in river‐floodplain ecosystems.

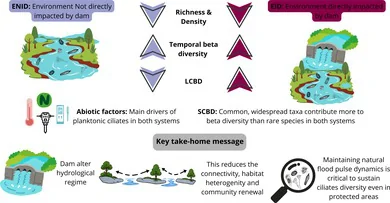

## 1 Introduction

Aquatic ecosystems, particularly floodplains, are highly dynamic environments characterised by significant habitat heterogeneity, high species diversity, and elevated productivity (Thomaz et al. 2007). The hydrological regime is the key factor influencing ecological functionality and biodiversity patterns in river‐floodplain systems (Neiff 1990). This regime is responsible for inducing seasonal alterations in water levels, modifying connectivity between environments, regulating the quantity and quality of available habitats, and altering limnological characteristics (Junk et al. 1989). These changes directly affect the organisation of aquatic communities, as multiple groups of aquatic organisms are associated with the ecosystem multifunctionality (i.e., the capacity of ecosystems to support multiple ecological functions simultaneously) (Moi et al. 2022). Natural flood events are also essential for maintaining environmental variability, high productivity, nutrient cycling, and other ecosystem services (Agostinho, Gomes, et al. 2004; Diniz et al. 2023). However, these essential environments are increasingly impacted by anthropogenic activities, including land‐use change, pollution, wildfires and dam construction (Agostinho, Gomes, et al. 2004). Dams change the natural hydrological regime, affecting the physical, chemical and biological environment, interfering directly or indirectly in the habitat structure, community composition and functional aspects of the system, mostly impacting species whose life cycles depend on these fluctuations and the connectivity between habitats (Agostinho, Gomes, et al. 2004).

Regarding community structuring, it is common to assess local factors influencing communities by examining α diversity, typically expressed as species richness and density (Magurran 2013). However, beyond the local scale, the regional scale—associated with γ diversity—represents the total number of species within a given region (i.e., the regional species pool), while β diversity reflects species turnover among environments (Magurran 2013). The β diversity has gradually become a central focus for ecologists over the last few decades, as it is a fundamental approach to understanding biodiversity patterns and guiding conservation practices (Legendre and De Cáceres 2013; Socolar et al. 2016).

The β diversity is closely linked to gradients in α diversity and both are shaped by the effects of environmental selection (Soininen et al. 2018). However, β diversity offers a more dynamic perspective on biodiversity patterns than α diversity metrics alone (Soininen et al. 2018). It plays a central role in understanding the ecological processes that shape biodiversity patterns across multiple spatial scales (Leibold et al. 2004) and acting as a sensitive indicator of community shifts (Magurran and Henderson 2010). In this way, β diversity has increasingly been used to gain deeper insights into the factors and mechanisms driving not only spatial but also temporal variation in communities (Legendre and Gauthier 2014). Several studies have reported the negative impacts of anthropogenic disturbances on α and β diversity in spatial and temporal scales (Bonecker et al. 2013; Diniz et al. 2023; Progênio et al. 2023; Zanon et al. 2024; Guimarães‐Durán et al. 2024).

According to Heino et al. (2024), compared to spatial β diversity, fewer studies have focused on aspects of temporal variation in community composition, known as temporal β diversity. However, conservation planning requires quantifying temporal variation in those communities' composition and the contributions that specific sites make to regional dynamics (Ruhí et al. 2017). Heino et al. (2024) define four unique aspects of β diversity that encompass the diverse applications of this concept in ecology and biodiversity management: (1) spatial β diversity, (2) temporal variation in spatial β diversity, (3) temporal β diversity, and (4) spatial variation in temporal β diversity. The integration of temporal and spatial approaches in ecological research is essential, as they offer a better understanding of community dynamics and highlight the need to consider both scales when addressing complex ecological questions (Lansac‐Tôha et al. 2021).

Over the past decade, a novel approach proposed by Legendre and De Cáceres (2013) has gained attention (Heino and Grönroos 2017). This method centers on the concept of ecological uniqueness and decomposes total β diversity into local contributions (LCBD) and species contributions (SCBD) to β diversity. As expected, assessments of ecological uniqueness have been recognised as valuable tools for guiding biodiversity conservation strategies (Haase et al. 2023). This information can help to better understand how anthropogenic impacts (e.g., the construction of dams) influences the distribution and density of different species (Zanon et al. 2024). According to Legendre and De Cáceres (2013), assessing the local contribution to β diversity (LCBD) across spatial or temporal scales provides valuable insight into diversity patterns and supports informed conservation and management strategies. These approaches have been widely applied across different geographic regions, ecosystems, and taxonomic groups (Heino and Grönroos 2017; Wang et al. 2020).

Among taxonomic groups, ciliate protists are key components of aquatic ecosystems. These organisms constitute a fundamental part of the pelagic compartment (Pauleto et al. 2009; Foissner et al. 1999; Asghar et al. 2025), contributing significantly to nutrient cycling by decomposing organic matter and regenerating nutrients for higher trophic levels in freshwater food webs (Garner et al. 2022). This role plays a crucial role in regulating the density and structure of planktonic communities through predation on microbial populations such as bacteria and algae (Meira et al. 2021), thereby enhancing their importance in ecosystem functioning, particularly regarding carbon metabolism and nutrient dynamics (Taniguchi and Menden‐Deuer 2023). Additionally, ciliate protists exhibit high sensitivity to changes in environmental conditions, such as temperature, salinity, seasonality, and hydrodynamics, making them excellent bioindicators of environmental quality and valuable tools for understanding ecological disturbances in aquatic systems (El‐Tohamy et al. 2024; de Matos et al. 2025).

Despite the recognised importance of ciliate protists in the dynamics of aquatic ecosystems, studies on the temporal variation, especially long‐term studies, are still scarce (Guimarães‐Durán et al. 2024), which limits the understanding of seasonal and pluriannual variations and the cumulative effects of environmental impacts on these organisms. Therefore, long‐term studies are of the utmost importance for understanding ecological variations in aquatic communities (Abdullah Al et al. 2022), especially in dammed environments, such as the Upper Paraná River, for effectively understanding and mitigating the impacts of damming on aquatic communities.

Here, we focused on spatial variation in temporal β diversity, aiming to assess long‐term trends in planktonic ciliate community structure over a 14‐year period across contrasting floodplain subsystems with different degrees of direct exposure to dam regulation. We hypothesised that environments not directly impacted by dams (ENID) exhibit distinct spatiotemporal patterns of community structuring compared to dam‐impacted environments. Based on this, we formulated the following predictions: **P1**—Protist richness and density will decline over time in dam‐impacted environments but remain comparatively stable in ENID. **P2**—We expect that river damming will result in stronger alterations in species composition, leading to higher temporal β diversity in impacted environments compared to non‐impacted ones. **P3—**Higher LCBD values will be found in the environments not directly impacted by dams (ENID), as they harbour more heterogeneous habitats. Moreover, these environments may support species with low frequency or restricted distribution. However, their contribution to β diversity (SCBD) will depend on how unevenly they are distributed across sites, and not solely on their rarity. **P4**—Finally, we expect that the temporal component will be a major driver of ciliate community structure in the environment impacted by dam (abbreviated throughout this work as EID), as damming reduces the amplitude of natural hydrological variation. In contrast, in the environment not impacted by dams (abbreviated as ENID), communities are expected to be primarily structured by local factors, such as abiotic and biotic (predation and resource) conditions (Table 1).

**TABLE 1:** Hypotheses, predictions and proposed ecological mechanisms underlying patterns of planktonic ciliate biodiversity in Upper Paraná floodplain.

Question	Prediction and current references	Ecological mechanisms and applicable theories
P1. How do protist richness and density change over time under contrasting dam influence?	Richness and density are expected to decline in EID but remain comparatively stable in ENID. Magurran and Henderson (2010); Bonecker et al. (2013); Diniz et al. (2023)	Reduction of flood pulses and connectivity compromises recruitment and dispersal of sensitive species. Flood pulse concept—Junk et al. (1989) Metacommunity theory—Leibold et al. (2004) Cumulative effects of disturbances—Odum (1969)
P2. Does damming result in greater temporal β diversity in planktonic communities?	Impacted environments should exhibit greater temporal β diversity due to successive changes in composition. Legendre (2019); Diniz et al. (2023); Zanon et al. (2024)	Chronic disturbances cause successive reorganisations of communities, increasing temporal variation. Species replacement theory—Whittaker (1960) Disturbance theory—Odum (1969); Connell (1978) Metacommunity—Leibold et al. (2004)
P3. Do environments not directly impacted by dams (ENID) have greater ecological exclusivity (LCBD) and a higher occurrence of rare species?	Unimpacted environments, being more heterogeneous, are expected to present higher LCBD values and harbour species with restricted distribution. Legendre and De Cáceres (2013); Heino and Grönroos (2017); Haase et al. (2023)	Heterogeneous environments favour specialist species and local exclusivity. The contribution to β diversity (SCBD) depends on spatial variability in density. Theory of environmental heterogeneity—MacArthur (1961) Partitioning of β diversity—Legendre and De Cáceres (2013) Niche theory—Hutchinson (1957)
P4. What factors structure the composition of communities in impacted and environments not directly impacted by dams (ENID)?	In impacted environments, a greater influence of the temporal component is expected, while local factors (abiotic and biotic) would predominate in environments not directly impacted by dams (ENID). Legendre and Legendre (1998); Simões, Dias, et al. (2013); Simões, Lansac‐Tôha, and Bonecker 2013; Leclerc et al. (2023)	The loss of hydrological variability favours temporal filters over local filters. Resources and abiotic variables structure communities. Environmental filter theory—Keddy (1992) Niche theory—Hutchinson (1957) Structuring by environmental gradients—Whittaker (1978)

## 2 Methods

### 2.1 Study Area and Data Collection

**FIGURE 1:**
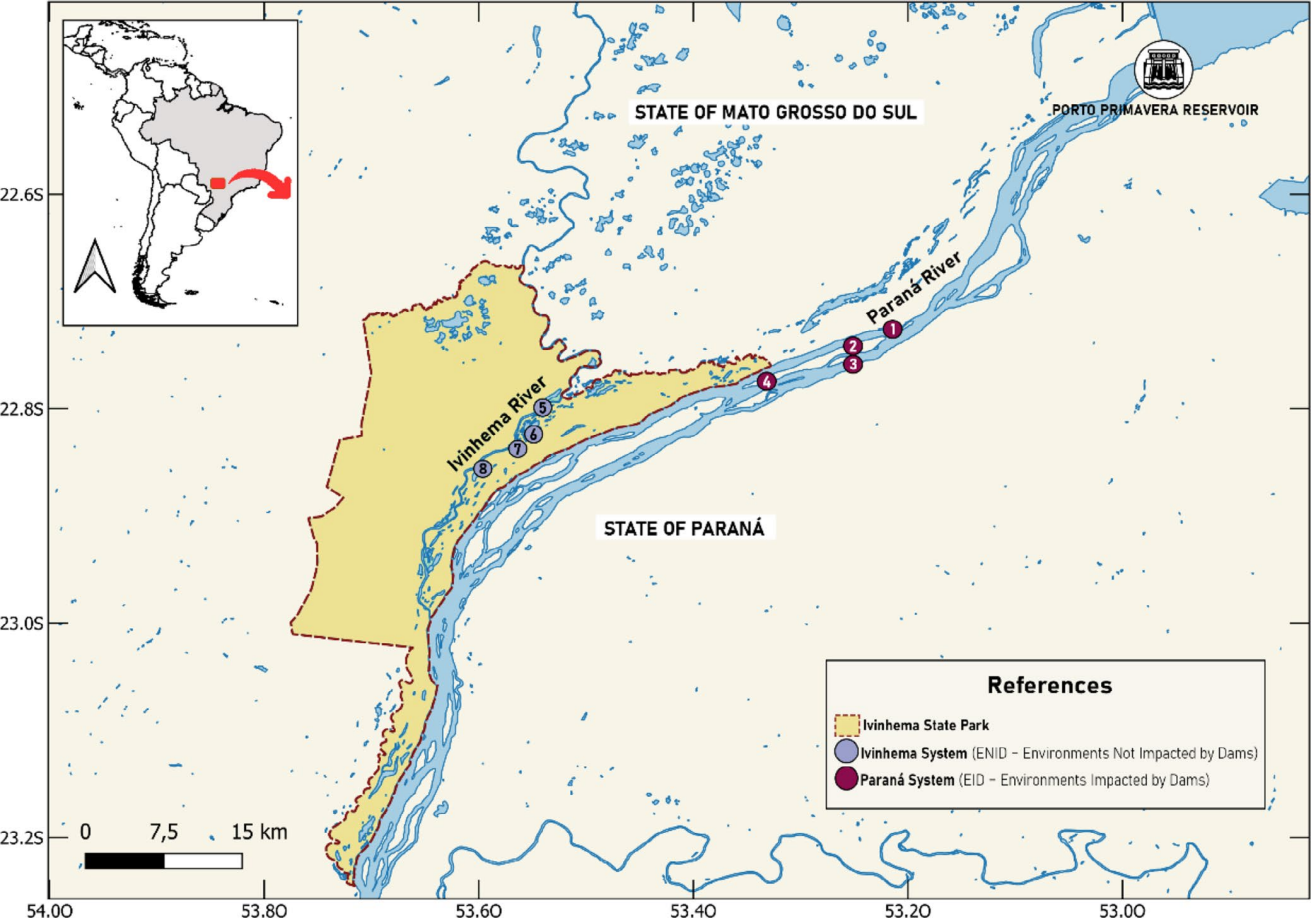
Map of the Upper Paraná River floodplain showing the sampled environments across two systems: The Paraná System (EID—Environments Impacted by Dams, represented by the dark purple colour) and the Ivinhema System (ENID—Floodplain subsystem without direct dam regulation but hydrologically connected to the Paraná River, represented by the colour light purple). The sampling sites include main rivers, secondary channels, open lakes (connected to the main river), and isolated lakes (isolated from the main river). Locations were monitored from 2010 to 2024 for temporal assessments of ciliate protist communities. In the map of South America, Brazil is highlighted in grey, and within that country, the study area is highlighted in the pink quadrant. 1 = Garças lake; 2 = Pau véio backwater; 3 = Paraná river; 4 = Fechada lake; 5 = Ivinhema river; 6 = Patos lake; 7 = Ventura lake; 8 = Curutba channel.

The Upper Paraná River floodplain (22°40′–22°50′ S, 53°10′–53°40′ W) is located between the Porto Primavera Reservoir (São Paulo State; 22°37′ S, 53°6′ W) and the Itaipu Reservoir (Paraná State, Brazil; 23°55′ S, 54°8′ W) (Figure 1), representing the last undammed stretch of this river. For satellite images of this stretch, see Supporting Information S1.

The Porto Primavera Reservoir represents one of the main hydropower developments for the hydrologic regulation of the upper Paraná River. Its operation, began in 1999, consolidated a historical process of dam construction in the basin, which already included several upstream reservoirs, resulting in significant changes in the hydrological regime, water quality, and the ecologic dynamic of the river‐floodplain system (Agostinho, Thomaz, and Gomes 2004; Agostinho, Gomes, et al. 2004; Stevaux et al. 2009; Roberto et al. 2009). Flow regulation by the reservoir has substantially altered the natural variability of discharges, reduced the amplitude of annual floods and increased the frequency of lower‐intensity hydrological pulses. After impoundment, water‐level variation generally ranged from about 1 to 7 m, with a predominance of low‐magnitude flooding events and rare occurrences of more intense floods (Rocha 2010). In addition, rapid sub‐daily fluctuations in discharge and water level have been recorded as a direct result of hydropower operation, influencing ecological processes and the structure of aquatic habitats in the adjacent floodplain (Agostinho et al. 2008).

This area is protected by three conservation units: the Protected Area of the Islands and Floodplains of the Paraná River, the Ilha Grande National Park, and the Ivinhema State Park (Agostinho, Thomaz, and Gomes, et al. 2004; Agostinho Gomes, et al. 2004). Its habitats hold high ecological importance due to their heterogeneity, comprising rivers, secondary channels, and lakes with varying degrees of connectivity (Souza‐Filho 2009), which result in high biodiversity of both aquatic and terrestrial organisms (Thomaz et al. 2007).

These environments are characterised by seasonal hydrological periods, alternating between flooding and drought events (Lansac‐Tôha et al. 2019). However, the construction of upstream dams along the Paraná River has significantly altered the connectivity between habitats (Agostinho et al. 2008; Lansac‐Tôha et al. 2019), affecting hydrological attributes and the dynamics of downstream communities (Thomaz et al. 2007; Agostinho et al. 2008, 2009; Braghin et al. 2018; Alves et al. 2021). Moreover, long‐term studies in the floodplain indicate that, due to reservoir retention, the Paraná River faces a substantial reduction in nutrient availability downstream of the dams (Roberto et al. 2009; Souza‐Filho 2009). In contrast, the Ivinhema River, free from dams along its course, maintains higher hydrological connectivity and presents distinct characteristics, such as high organic matter content, elevated turbidity, and higher nutrient concentrations (Roberto et al. 2009).

The basins include rivers and channels characterised by higher flow velocity and greater depth compared to lakes. Open lakes have a direct connection with the rivers, which facilitates greater exchange of nutrients and resources. In contrast, isolated lakes exhibit lower flow velocity and reduced nutrient renewal, being generally connected to the rivers only during flood periods. To visualise the characteristics of environments in each system, see Supporting Information S2.

### 2.2 Sampling and Environmental Characterisation

The study spanned 14 years (2010–2024) and comprised 42 sampling campaigns conducted at eight sites distributed across two floodplain subsystems: four in the Paraná River system (EID) and four in the Ivinhema River system (ENID). Sampling was generally conducted four times per year, covering two hydrological periods: the dry period, represented by June and September, and the rainy period, represented by December and March. Due to restrictions imposed by the COVID‐19 pandemic, only one sampling campaign was conducted in each of the years 2020 and 2021 (March 2020 and December 2021, respectively). The sampling design was structured to ensure comparability and representativeness between systems. In each subsystem (EID and ENID), we selected the same four types of aquatic environments: main river, secondary channel, open lake, and closed lake. These environments represent a well‐established hydrological gradient in river–floodplain systems, ranging from highly lotic and permanently connected habitats to lentic environments with reduced or episodic connectivity. Thus, the number and type of environments were balanced between systems, allowing differences in community patterns to be interpreted primarily in relation to dam impact rather than to differences in habitat composition. This design ensures that each system is comparably represented across major environmental categories characteristic of the Upper Paraná River floodplain.

Water‐level data were obtained from the Paraná River (EID), where long‐term hydrometric monitoring is conducted. The Paraná River is considered the main hydrological driver of the Upper Paraná River floodplain and can influence adjacent environments, including those associated with the Ivinhema subsystem Thomaz et al. (2004) and Train and Rodrigues (2004). However, the Paraná and Ivinhema subsystems are not hydrologically independent, as connectivity between them may occur during high‐water periods. Therefore, Paraná River water level was used as an indicator of regional hydrological dynamics rather than as evidence of subsystem independence. This approach is consistent with previous studies in the Upper Paraná River floodplain, which have demonstrated that hydrological connectivity varies temporally and spatially across subsystems.

At each site, subsurface water was collected from the central region of the environment using polyethylene bottles and subsequently transferred to five‐litre containers (Oliveira et al. 2024). Samples were stored in coolers and transported to the laboratory, where they were filtered through a 10‐μm plankton net and concentrated to a final volume of 100 mL for in vivo community analysis (Madoni 1984). Quantitative analyses were performed using single‐channel micropipettes to obtain ten 100‐μL aliquots (1 mL total), which were counted on microscope slides following Madoni (1984). To catalogue rare species not detected in quantitative counts, qualitative analyses were performed using a 1‐mL Sedgewick–Rafter chamber. Both analyses were conducted under an optical microscope (Olympus, model CX31) at 40×, 100×, and 400× magnifications. Species were identified to the lowest possible taxonomic level using specialised literature (Foissner and Berger 1996; Foissner et al. 1999, 2003).

Simultaneously with plankton sampling, a comprehensive set of environmental variables was measured following (American Public Health Association (Apha) 1926). These included water and air temperature (°C), pH, conductivity (μS cm^−1^), dissolved oxygen (mg L^−1^ and % saturation), wind speed (m s^−1^), water depth (m), turbidity (NTU), Secchi depth (m), total suspended matter (mg L^−1^) and its inorganic and organic fractions, alkalinity (Eq L^−1^), and *chlorophyll‐a* (μg L^−1^). Nutrient concentrations were also determined, including total nitrogen (μg L‐^1^), nitrate (N‐NO_3_^−^, μg L^−1^), ammonium (N‐NH_4_^+^, μg L^−1^), total phosphorus (μg L^−1^), and orthophosphate (P‐PO_4_^3−^, μg L^−1^). Detailed procedures for each variable are available in Santana et al. (2017).

#### 2.2.1 Phytoplankton and Zooplankton

Zooplankton samples were taken using a motorised pump to filter 1000 L of water through a plankton net (68 μm). The collected material was stored in polyethylene bottles and fixed in 4% formaldehyde (buffered with calcium carbonate). The samples were concentrated in a known and variable volume (75–300 mL), considering the number of organisms and the amount of sediment in the sample, which can make it difficult to visualise specimens in the sample. Before the analysis, the samples were stained with Bengal Rose to improve the visualisation of the organisms. The density of the four main zooplankton groups considered here (testaceous protozoa, rotifers, cladocerans, and copepods) was estimated by counting, in Sedgewick–Rafter chambers, five sub‐samples, 1.5 mL (total 7.5 mL), obtained with a Hensen–Stempel pipette, with the final density results expressed in ind m^−3^. Since the subsampling method is not sufficient to provide satisfactory results of species richness, after counting the sub‐samples, a qualitative analysis sample was carried out. Samples with very low densities were analysed in full. We consider species richness as the number of species present in each sample.

Phytoplankton samples for quantitative analyses (i.e., richness and density calculations) were collected directly with bottles and fixed with 1% acetic Lugol solution. With an inverted microscope and 400× magnification, we performed the taxonomic quantitative and qualitative analysis of the samples. The counting of individuals (i.e., cells, coenobium, colonies, and filaments) followed Utermöhl (Utermöhl 1958), with the previous sedimentation of the samples, considering at least 3 h for each millilitre of sample. Species density was calculated according to APHA (American Public Health Association (Apha) 1926), and the results were expressed in individuals per millilitre (ind mL^−1^).

## 3 Data Analysis

Water levels were measured daily by a gauge installed at the field in the EID system. This gauge, installed in the field, allowed for remote monitoring of water levels, with this water level being observed at the advanced research base of the Research Center in Limnology, Ichthyology and Aquaculture, at the State University of Maringá. To summarise the environmental variations (based on abiotic data) and analyse the changes between EID and ENID, we carried out a principal component analysis (PCA). All explanatory variables were screened for multicollinearity by applying variance inflation factors (VIF), and variables with VIF values greater than 7 were excluded from the analysis (Legendre and Legendre 1998). The environmental characterisation revealed collinearity between the predictor variables mean surface temperature (MST) and dissolved oxygen (mg L^−1^), which had high variance inflation factors (VIF), and was not used in the PCA (or in any subsequent pRDA models). After removing the MST variable, the remaining variables showed low collinearity, with VIF values below 2 and pair correlation coefficients ranging from 0.0005 to 0.569. We used a multivariate dispersion homogeneity test (PERMDISP) to test environmental differences of PCA, considering *p* significant values those < 0.05.

To test the **P1** prediction, we used generalised additive models (GAMs) to compare temporal trends in protist richness and density between EID and ENID. The GAMs were implemented using the ‘*mgcv*’ package in R (Wood 2016), separately for species richness and density. We adopted a negative binomial distribution with a log link function for both variables to account for overdispersion and the discrete nature of the data. GAMs extend the classical generalised linear model (GLM) framework by replacing the linear predictor with a non‐parametric smoothing function, which allows for the modelling of potentially nonlinear temporal patterns (Wood 2006).

To address our second prediction (**P2**), which proposed that river damming would lead to stronger alterations in species composition, resulting in higher temporal β diversity in EID compared to ENID, we evaluated both temporal and regional variation in community composition. Temporal β diversity was assessed by comparing the initial sampling event with each subsequent event at each site, allowing us to quantify how much the community structure changed. In this context, an increase in dissimilarity over time is interpreted as evidence of a directional shift in community composition (Legendre 2019). We used the TBI (Temporal β Index) function from the adespatial package (Dray et al. 2019) to perform these calculations. Additionally, temporal β diversity was calculated across all sites simultaneously at each time point to capture broader regional patterns of temporal variation. To evaluate temporal trends, the resulting TBI values were analysed using a beta regression model suitable for continuous data bounded between 0 and 1 (Ferrari and Cribari‐Neto 2004), with TBI as the response variable and time as the predictor, fitted with a logit link in the betareg package in R.

To address our third prediction (P3), that LCBD would be higher in non‐impacted than in impacted environments due to greater habitat heterogeneity, we calculated local (LCBD) and species (SCBD) contributions to β diversity following Legendre and De Cáceres (2013). LCBD quantifies the ecological uniqueness of each site, whereas SCBD measures the contribution of each species to among‐site β diversity (e.g., Heino and Grönroos 2017; Liu et al. 2023). These metrics were used to identify sites and taxa that disproportionately contribute to spatial variation in community composition between impacted (EID) and environments not directly impacted by dams (ENID).

LCBD and SCBD were calculated following Legendre and De Cáceres (2013) using Jaccard distance and the *beta.div* function from the *adespatial* package. LCBD was further related to species richness and to an index of environmental uniqueness, defined as the Euclidean distance of each site to the multivariate centroid in standardised environmental space. To explore how the number of sites occupied by each species was related to its SCBD value, we fitted beta regression models including linear and quadratic terms for occupancy and selected the best‐fitting model based on AIC criteria. All regression models with LCBD or SCBD as response variables were fitted using beta regression (*betareg* package; Cribari‐Neto and Zeileis 2010), appropriate for variables bounded between 0 and 1. Environmental variables were standardised with the decostand function from the *vegan* package (Oksanen et al. 2025), and all figures were created using *ggplot2* (Wickham 2016). Differences in SCBD values between systems and environment types were tested using Kruskal–Wallis tests. Only *p*‐values lower than 0.05 were considered significant.

To address our fourth prediction (**P4**), in which we hypothesised that the temporal component would be the primary driver of ciliate community structure in EID due to reduced hydrological variability from damming, while in ENID communities would be structured mainly by local abiotic and biotic factors, we performed partial redundancy analysis (pRDA). The response matrix consisted of ciliate species composition data, transformed using the Hellinger method to accommodate the high prevalence of zeros. As explanatory matrices, we used four groups of variables. The environmental set included the variables previously selected by VIF (water temperature, dissolved oxygen in percentage saturation, pH, conductivity, Secchi disc depth, turbidity, total nitrogen, total phosphorus, phosphate, and MSI and MSO variables). The temporal set comprised a categorical variable describing the hydrological period (Dry vs. Rainy). The predation set consisted of presence–absence data of zooplankton species, and the resource availability set consisted of presence–absence data of phytoplankton species. Temporal, predation, and resource matrices were treated analogously to spatial explanatory variables in classical pRDA frameworks, as they represent structured gradients potentially influencing community composition.

Variable selection described (such pH, dissolved oxygen, total phosphorus, and the others) within each explanatory group was performed through forward selection (with 999 permutations and *α* = 0.05), using the *ordiR2step* function in the vegan package (Oksanen et al. 2025). Adjusted *R* ^2^ values were retained as indicators of the relative explanatory power of each fraction, as they are not inflated by the number of variables and therefore allow for more robust comparisons (Peres‐Neto et al. 2006).

From this variation partitioning, we estimated the total variance explained by each group of variables, the shared fraction between groups, and the pure fractions of each group (after controlling for the influence of the others). The significance of each pure fraction (excluding the shared component, which cannot be tested) was evaluated by permutation tests (999 permutations, *α* = 0.05). These procedures allowed us to identify the main structuring processes of ciliate communities in impacted versus non‐impacted floodplain environments, distinguishing the roles of local abiotic conditions, biological interactions (predation and resources), and temporal variability.

Because the COVID‐19 pandemic restricted sampling to a single campaign in 2020 and 2021, the resulting time series contains irregular intervals, which our analytical framework accommodates. In the GAMs, sampling time was modelled as a continuous covariate (decimal year) using regression patterns, which do not assume equally spaced observations and whose smoothing prevents sparsely sampled periods from exerting undue leverage, widening the confidence intervals where data are fewer. For the TBI, each campaign was compared pairwise with the initial (baseline) campaign, so that missing intermediate campaigns do not bias any individual dissimilarity value. To further verify robustness, all temporal analyses were additionally re‐run with the 2020 and 2021 campaigns excluded. The results were qualitatively unchanged, and we therefore retained the complete series in the main analysis to preserve the continuity of the long‐term record.

## 4 Results

**FIGURE 2:**
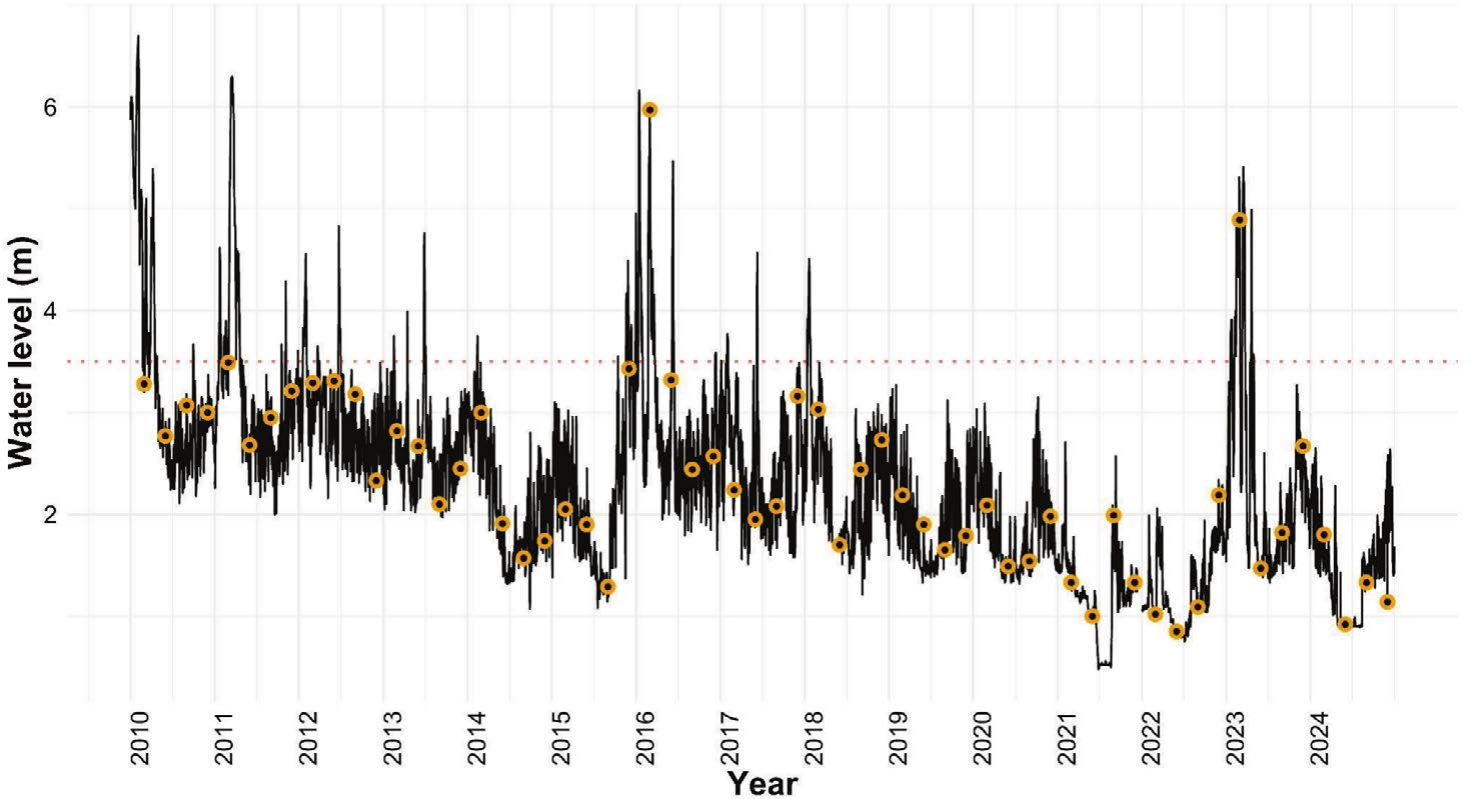
Daily water‐level fluctuations in the Upper Paraná River from 2010 to 2024, based on data obtained from the Field Station of Maringá State University. Yellow circles indicate the sampling dates. The dashed horizontal line represents the 3.5‐m water level associated with potential hydrological connectivity between the EID and ENID systems. Daily measurements of water‐level fluctuation in the Upper Paraná River (data obtained at the Field Station of Maringá State University) from 2010 to 2024. The dashed line running through the graph represents the 3.5‐m level that allows for the hydrological connection between the EID and ENID systems.

Throughout the study period (2010–2024), the water level of the Paraná River exhibited strong seasonal and interannual variation, though with a general trend of reduction (Figure 2). It was observed that in the initial years of monitoring, more frequent and intense floods were recorded, with peaks exceeding 6 m and surpassing the 3.5 m limit. This 3.5 m level is significant because, above it, the Paraná River begins to directly influence the ENID (Thomaz et al. 2004). In more recent years, lower water levels prevailed, with less frequent events above 3.5 m and more restricted amplitudes of variation.

**FIGURE 3:**
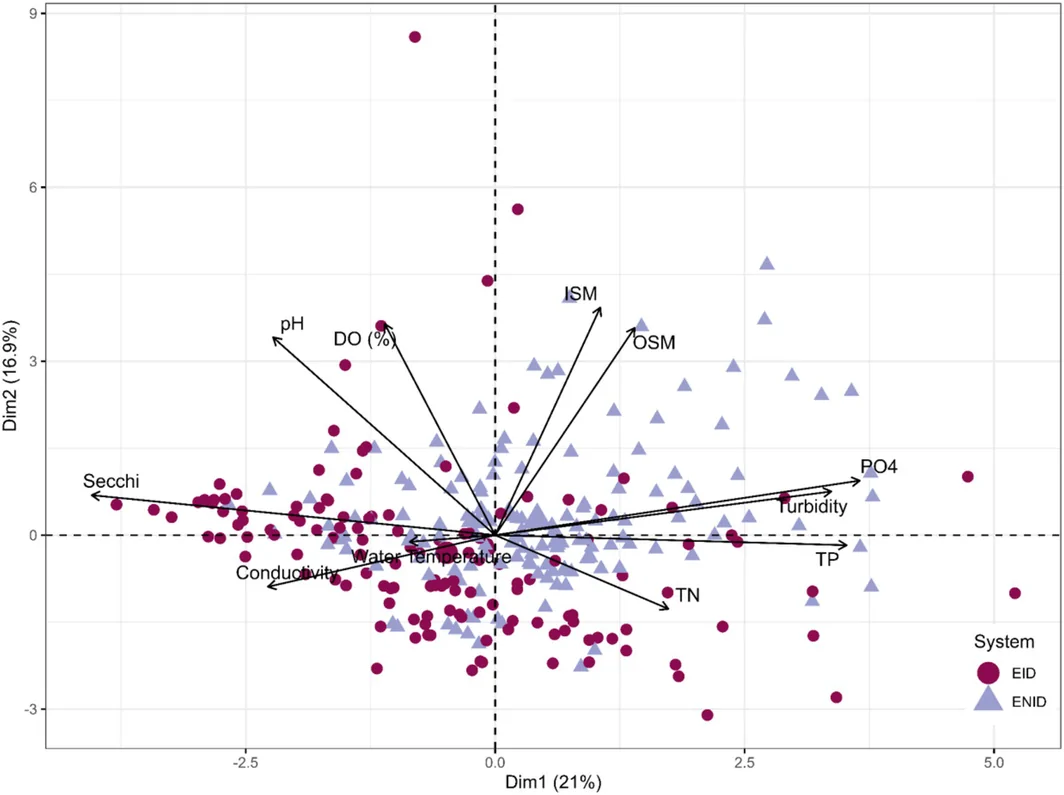
PCA biplot based on environmental variation represented in the first two axes of a principal component analysis, which discriminates between the EID and ENID environments. PO_4_ = phosphate; TN = total nitrogen; TP = total phosphorus; DO (%) = dissolved oxygen (percentage saturation); ISM = inorganic suspended matter; OSM = organic suspended matter. PCA biplot based on environmental variation represented by the first two axes of the principal component analysis, discriminating between the EID and ENID environments. Dashed reference lines indicate zero on both PCA axes. PO4 = phosphate; TN = total nitrogen; TP = total phosphorus; DO (%) = dissolved oxygen (percentage saturation); ISM = inorganic suspended matter; OSM = organic suspended matter.

Principal component analysis (PCA; Figure 3) confirmed the consistency of the environmental data structure, with the first two axes explaining 21% and 16.9% of the total variance, respectively. The PCA biplot indicated no strong segregation between the systems over time, suggesting that overall environmental heterogeneity remained relatively stable throughout the monitoring period.

When analysing the environmental variables between the systems, Axis 1 shows a positive correlation with MSI, MSO, PO_4_, and turbidity. The axis shows greater segregation between the systems, with the ENID system contributing more significantly to this axis, clearly separating it from the EID system. Axis 1 also shows a strong negative correlation with pH, Secchi disc, and dissolved oxygen (in percentage).

For Axis 2, we observed a negative correlation with Total Phosphorus (TP), Total Nitrogen (NT), conductivity, and water temperature. The EID system is widely distributed along this axis; thus, among the axes of the PCA, the measured environmental variables showed greater variations for the EID system.

**FIGURE 4:**
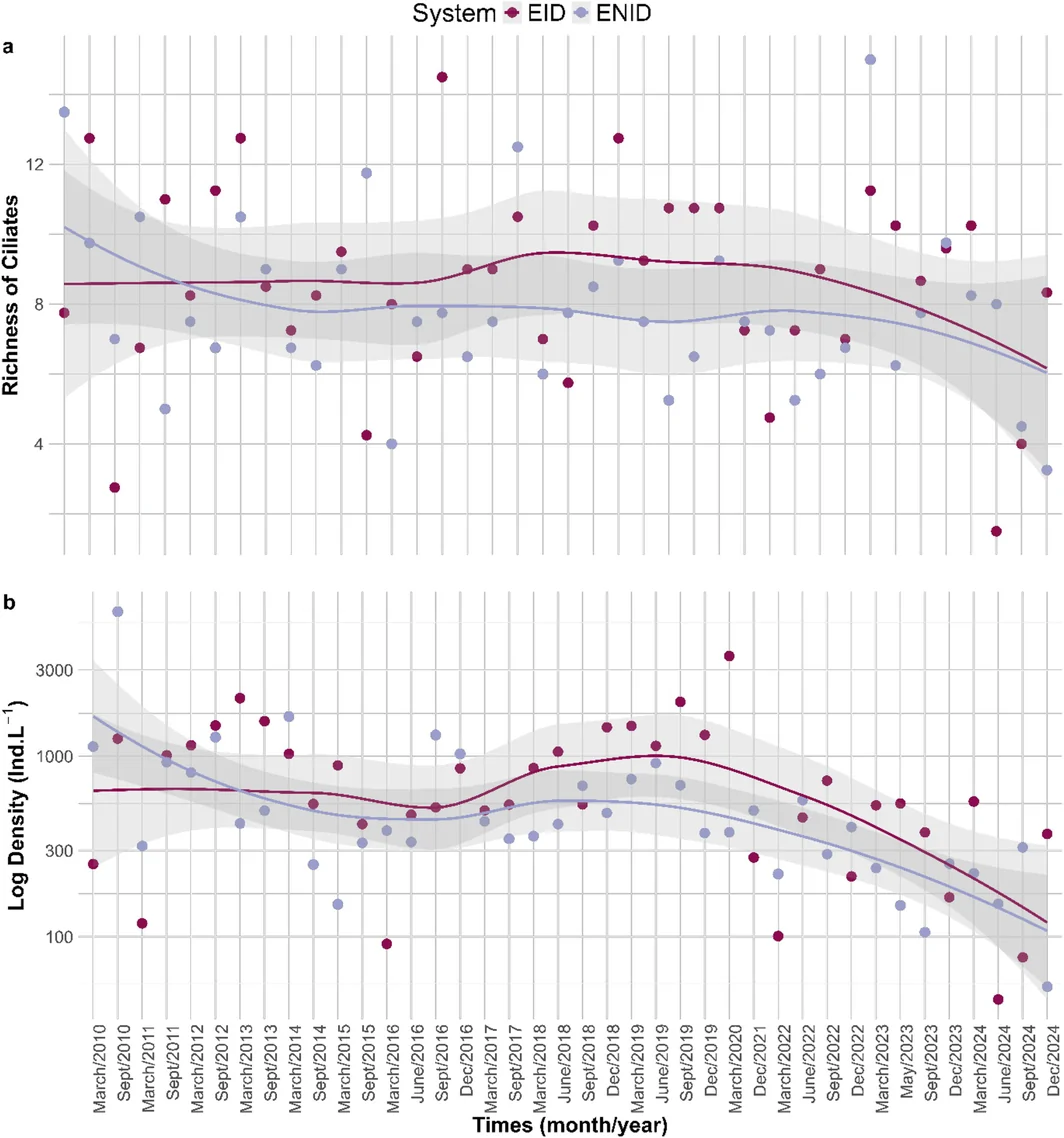
Smoothing curves (solid lines) obtained from generalised additive models (GAMs) showing temporal fluctuations in (a) taxon richness (number of species) and (b) density (individuals L^−1^) of protozooplanktonic groups in the Ivinhema (ENID—light purple) and Paraná (EID—dark purple) systems, across 42 sampling campaigns, from March 2010 to December 2024. The observed values for each campaign are represented by the coloured points, while the grey shaded regions surrounding each line denote the 95% confidence intervals of the fitted smooth terms.

In total, 217 ciliate morphospecies were recorded, with 191 in the EID system and 161 in the ENID system (S1; S2). Both EID and ENID showed a general trend of reduction over the study period (2010–2024) (Figure 4a,b). Generalised Additive Models (GAM) time series analysis revealed a declining pattern for both species richness and density in both systems. In the ENID, richness was high in the initial years, followed by a sharp drop until 2012, then relative stability, and a further decline from 2019 (Figure 4). Similarly, in the EID system, richness initially decreased, then increased between 2013 and 2019, and then fell again in more recent years.

**FIGURE 5:**
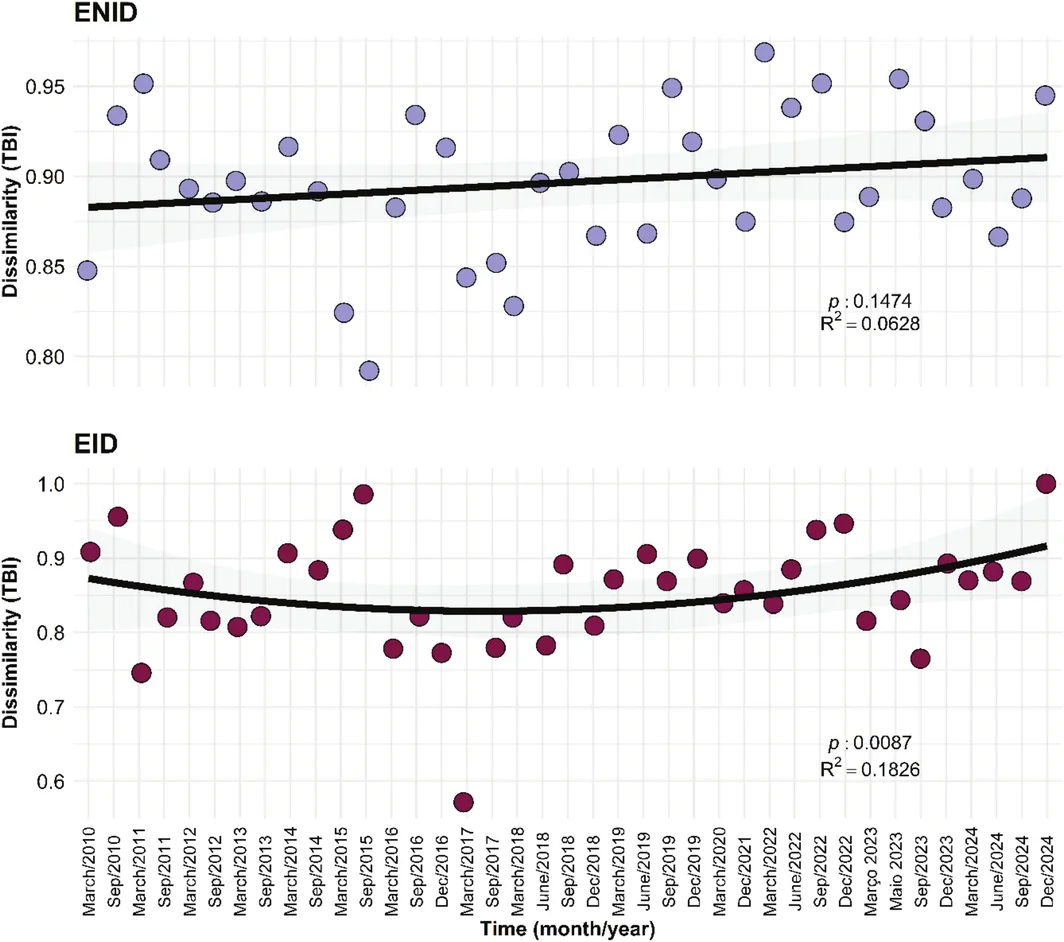
β regression graphs between local β diversity (dissimilarity) and time (sampling period). (a) and (b) show the comparisons of the temporal variation between the samples compared to the first over the period 2010–2024. Beta regression between local β diversity (dissimilarity) and time (sampling period) for the ENID and EID systems from 2010 to 2024. The panels are identified directly as ENID and EID. Coloured circles represent the observed local β‐diversity values for each sampling period.

The fitted model for Temporal Beta Index for EID system, incorporating sequential inter‐campaign comparisons, revealed a significant quadratic trend in ciliate community composition over time (*p* = 0.008; *R* ^2^ = 0.182), suggesting a nonlinear pattern of temporal dissimilarity variation. In contrast, no significant temporal effects were detected for ENID (*p* = 0.147; *R* ^2^ = 0.062), indicating temporal stability in community composition (Figure 5). This lack of a significant temporal trend in ENID was robust to the reduced pandemic‐year sampling: when the single‐campaign years (2020 and 2021) were excluded, the ENID trend remained non‐significant and the EID trend remained significant, indicating that the temporal stability of ENID reflects a genuine ecological pattern rather than an artefact of low sampling effort.

**FIGURE 6:**
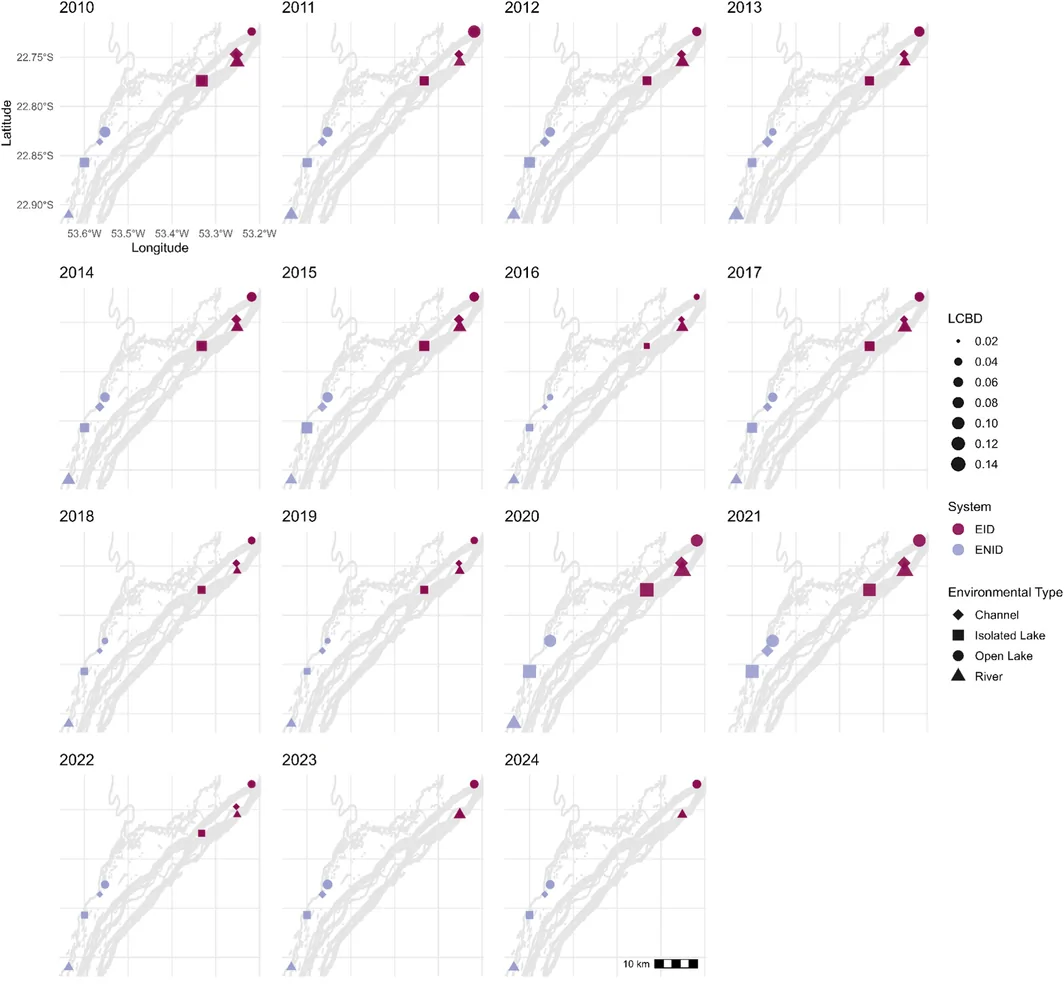
Local Contribution to β Diversity (LCBD) per year, across four environmental types in the ENID and EID systems. The larger the geometric figures, the higher LCBD values. It should be noted that the years 2020 and 2021 involved only a single sampling campaign due to the COVID‐19 pandemic, showing high values for this metric. However, we have retained these 2 years to maintain consistency with the 14‐year timeframe under evaluation.

The spatiotemporal analysis of Local Contribution to β Diversity (LCBD) values throughout the study period revealed high interannual variations in this index, with similar patterns between systems but some differences among the four environmental types within each system (river, channel, open lakes and isolated lakes; Figure 6). The high LCBD values observed in 2020 and 2021 in both systems resulted from single sampling events conducted in those years due to the COVID‐19 pandemic. To confirm that this sampling limitation did not affect our results, we re‐ran LCBD analysis excluding the 2020 and 2021 campaigns. The outcomes were qualitatively identical to those obtained with the complete dataset, and the spatial LCBD pattern described below was preserved. We therefore retained these years in the main analyses and interpret the 2020–2021 LCBD values only in light of this limitation.

Over the study period, the highest LCBD values were predominantly concentrated in lacustrine environments (open lakes and isolated lakes) and channels of the EID system. In contrast, environments within the ENID system generally exhibited lower LCBD values with reduced spatial and temporal variation.

**FIGURE 7:**
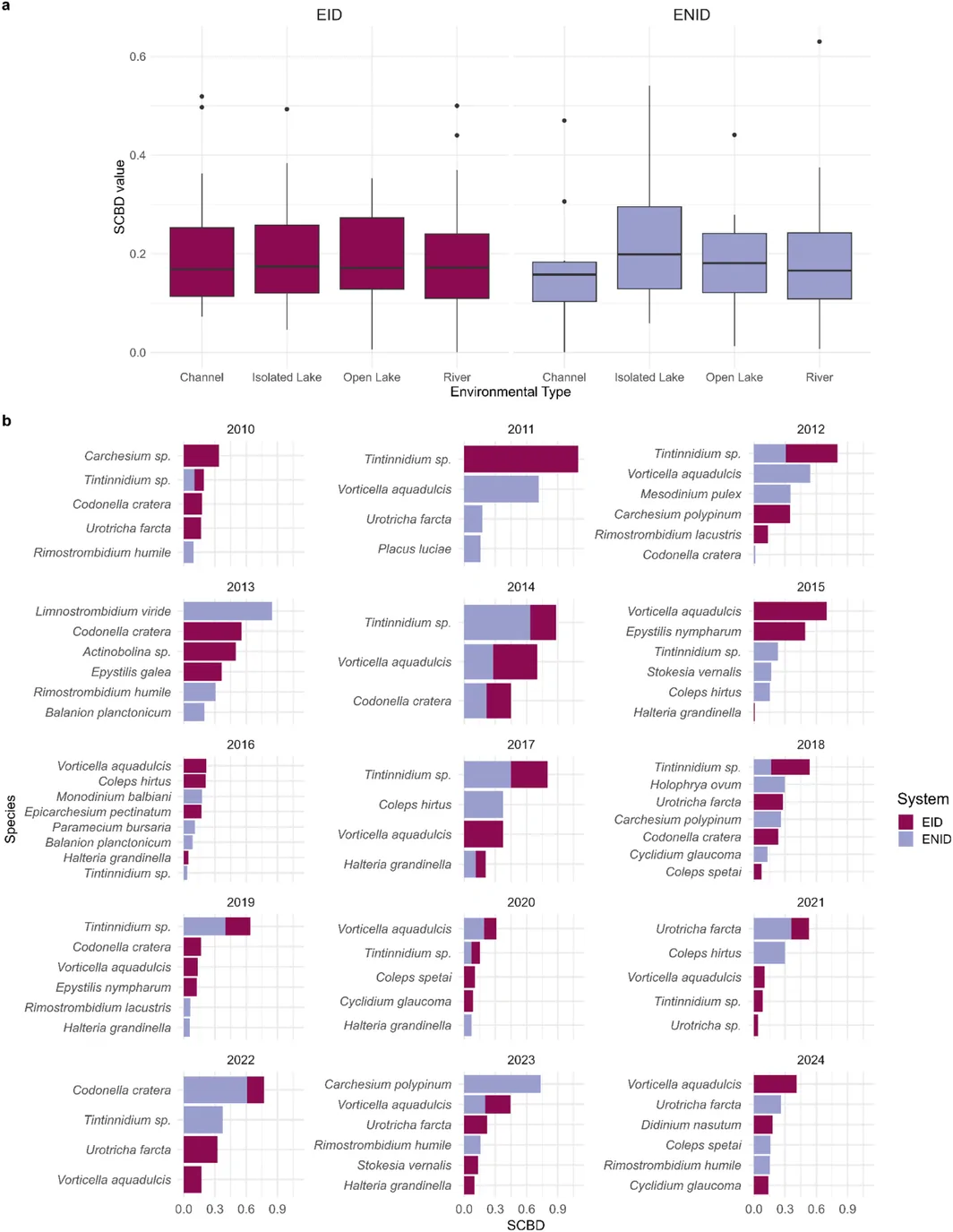
(a) boxplots of species contribution to β diversity (SCBD) across four environmental types (Channel, Isolated Lake, Open Lake, River) in the ENID and EID systems. (b) SCBD values for the most representative ciliate species (in descending order) in each year from 2010 to 2024, separated by system, considering all environmental types. Bar lengths represent individual species contributions.

We further conducted a Species Contribution to β Diversity (SCBD) analysis across the sampled periods and locations. The complete SCBD values for each environment and study year are provided in Supplementary Table S3. When comparing environmental types between systems, no significant differences in SCBD values were detected (Kruskal‐Wallis test, *p* > 0.05; Figure 7a).

Among the analysed species (Table S1; Figure 7b), *Limnostrombidium viride* attained the highest recorded SCBD value (SCBD = 0.630) in 2013 in the Ivinhema River, with a calculated density of 150 ind L^−1^. In the same year, *Actinobolina* sp. in the Paraná River exhibited a high SCBD (0.500), despite its low density (40 ind L^−1^).

Notably, *Vorticella aquadulcis*, observed in 2012 in Ventura Lake (an isolated lake in the ENID system), achieved a high species‐specific β diversity contribution (SCBD = 0.541). This species dominated the local community with a high density (20,450 ind L^−1^) during the sampling period. While *Tintinnidium* sp. emerged as a frequent species with consistently high SCBD values across the study. In 2011, it reached an SCBD of 0.519, reflecting its ecological relevance in shaping β diversity patterns.

**FIGURE 8:**
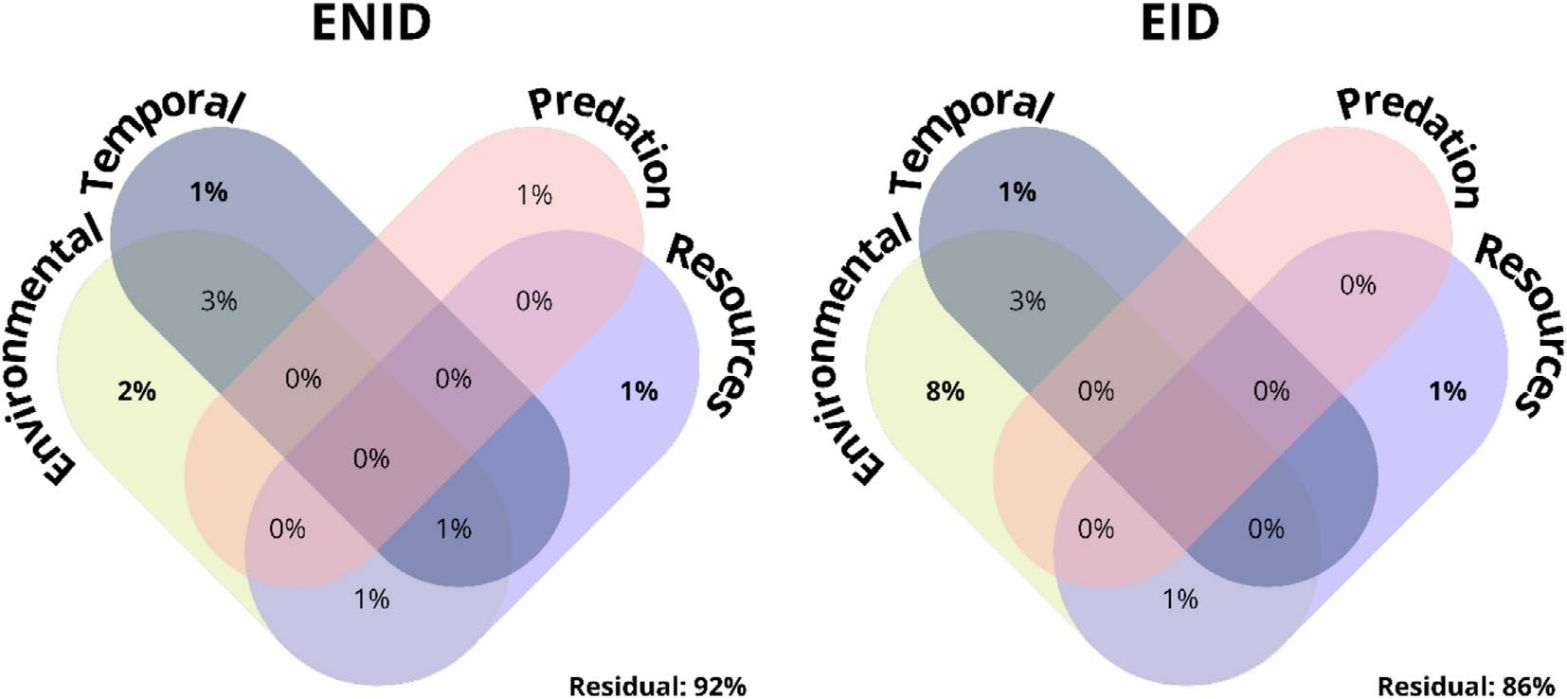
Partial redundancy analysis (pRDA) was performed using environmental and biotic data, with zooplankton and phytoplankton communities used as proxies for predation and resource availability, respectively, and bold values indicating statistically significant results (*p* < 0.05).

The overall pRDA model was significant for the EID system, explaining 12% of the total variation in ciliate protist community composition (*R* ^2^ = 0.12; *p* = 0.001). Among the evaluated factors, abiotic variables contributed the most (*R* ^2^ = 0.118; *p* = 0.001), indicating that physical and chemical environmental characteristics were key drivers of community structure. The temporal fraction was also significant (*R* ^2^ = 0.039; *p* = 0.001), suggesting relevant, though more modest, seasonal and interannual influences. The resource‐related component (phytoplankton) was significant but explained less variation (*R* ^2^ = 0.028; *p* = 0.009), whereas predation had no statistical significance (*p* = 0.491) (Figure 8).

Similarly, the pRDA model for the ENID system was globally significant (*R* ^2^ = 0.06; *p* = 0.001). Here, the environmental fraction explained 6.4% of the variation (*p* = 0.001), while the temporal fraction accounted for 3.5% (*p* = 0.001), reinforcing seasonal and abiotic effects on the community. As in the EID system, the resource component had a small but significant contribution (*R* ^2^ = 0.017; *p* = 0.025), whereas predation remained non‐significant (*p* = 0.079) (Figure 8).

## 5 Discussion

Contrary to our initial expectations, both systems (ENID and EID) exhibited similar responses regarding the structuring factors of ciliate communities. The variance partitioning analyses revealed that abiotic and temporal factors were the primary drivers of community composition variation in both systems, regardless of their disturbance levels. Among the biotic variables analysed, only resource availability (phytoplankton) had a significant though comparatively weaker effect.

These findings suggest that even in dam‐free environments within the floodplain, such as the ENID subsystem, which remain hydrologically connected to the Paraná River during high‐water periods, alterations in regional flood pulse dynamics may similarly influence planktonic community structure over time. This convergence in system responses highlights that conservation efforts should prioritise not only local habitat conditions, but also hydrological connectivity and seasonal variability, which are key elements for maintaining functional biodiversity in regulated floodplains.

Contrary to our predictions, we found no marked differences between subsystems with contrasting degrees of direct dam influence (EID and ENID) regarding temporal trends in ciliate protist richness and density. Both systems showed consistent declines over the study period, indicating that while dam impacts are relevant, they do not act in isolation in structuring planktonic communities. These results suggest the influence of additional regional‐scale factors, such as extreme climatic events and diffuse anthropogenic impacts, which may negatively affect diversity even in protected areas like ENID (Oliveira et al. 2018).

Damming directly alters water levels downstream of the Porto Primavera Dam on the Paraná River, heavily influenced by upstream reservoir operations (Bonecker et al. 2013). This leads to abrupt changes in physical and chemical parameters, affecting water quality and reducing environmental heterogeneity, which can drive biotic homogenisation downstream (Bredenhand and Samways 2009; Braghin et al. 2018; Guimarães‐Durán et al. 2024). Such processes contribute to declining β diversity (Wu et al. 2019). For ciliates, the observed reductions in richness and density may stem from lost hydrological connectivity due to persistently lower river levels and absent flood pulses, which limit species dispersal and planktonic inoculum exchange, a key process for species recruitment (Agostinho et al. 2016).

Although ENID is not directly regulated by dams, its protist diversity decline likely reflects regional pressures. Climate phenomena (e.g., El Niño/La Niña) can synchronize hydrological shifts across floodplain compartments (Agostinho et al. 2008; Bonecker et al. 2013; Alves et al. 2021; Pineda et al. 2022). Moreover, ENID's watershed faces agricultural, urban, and industrial activities, causing diffuse pollution, riparian degradation, and water quality changes (Agostinho et al. 2008, 2016). However, the most pronounced diversity loss occurred in EID, underscoring dams as cumulative stressors with more severe, persistent biodiversity impacts.

Unexpectedly, ENID exhibited patterns similar to those of EID. Although ENID is legally protected, it is not hydrologically isolated: the Ivinhema River is a tributary of the Paraná, and therefore indirectly influenced by the upstream dams that regulate the main river. These dam‐induced water‐level fluctuations propagate downstream, altering the timing and magnitude of ENID's natural flood pulses—processes essential for planktonic community dispersal, recruitment, and renewal (Oliveira et al. 2024). Artificial flow regulation is known to promote habitat homogenisation and reduce ecological connectivity, generating cascading effects on freshwater biota (Poff 1997; Bunn and Arthington 2002; Agostinho, Thomaz, and Gomes 2004). Thus, even areas with lower direct dam influence, such as ENID, may experience indirect hydrological pressures that constrain biodiversity, particularly in systems strongly shaped by flow regimes (Johnson et al. 2008). In addition to the reduction of the flood pulse in the study area, we observed a decline in hydrometric levels over the sampled period. The recorded decrease in water levels during the study may explain the variability in species density and richness across different environments, as demonstrated by Dias et al. (2017) for fish communities.

As predicted, the dam‐impacted system (EID) exhibited higher temporal β diversity, reflecting more intense and recurrent shifts in species composition over time. In contrast, the subsystem without direct dam regulation (ENID) showed greater temporal stability in ciliate community composition, despite displaying fluctuations primarily linked to progressive species loss as also shown by Guimarães‐Durán et al. (2024).

Although these systems differ fundamentally, the results suggest that dam effects may extend beyond directly regulated environments, indirectly influencing community dynamics in ENID (Graco‐Roza et al. 2021) through hydrological connectivity with the Paraná River. During flood periods, elevated water levels in the Paraná can modify limnological conditions in associated environments like ENID, acting as an environmental filter that regulates resource availability, organism dispersal, and ultimately community structure (Simões, Lansac‐Tôha, and Bonecker 2013). Thus, while regional pressures may affect both systems, dam operations exert stronger and more persistent effects on temporal compositional variability in EID.

The pronounced species turnover in EID likely stems from artificial hydrological alterations, which disrupt habitat availability, environmental heterogeneity, and flood pulses, all of which are key drivers of aquatic community structure (Petsch et al. 2021). In contrast, systems with higher hydrological integrity (e.g., ENID) tend to maintain greater ecological stability over time. Notably, the biotic homogenisation typically reported in dam‐regulated systems (Braghin et al. 2018) may manifest here not as reduced spatial diversity but as heightened temporal compositional instability. This pattern can reflect the replacement of sensitive species by taxa tolerant to dam‐induced conditions for other communities (Oliveira et al. 2018; Petsch et al. 2021). Critically, even with increased temporal β diversity, these communities may face reduced ecological resilience, as the observed fluctuations represent stress‐induced turnover rather than adaptive reorganisation.

Contrary to our hypothesis, the environments with lower direct dam exposure (ENID subsystem) did not show the highest LCBD and SCBD values, which would reflect greater species exclusivity and presence of rare taxa. Instead, the highest LCBD values occurred in the impacted system (EID), potentially explained by different mechanisms. High LCBD values may indicate either communities with rare species or dominance by common but uniquely distributed species (Hulot et al. 2021).

In the EID system, both processes appear to have occurred: on one hand, dam‐induced environmental changes, especially in the main river, promoted community structure simplification and occurrence of temporally exclusive species (Szabó et al. 2018; Farooq et al. 2024). Thus, over time, we observed a declining trend in LCBD values in this system, suggesting cumulative dam impacts and progressive habitat loss (Guimarães‐Durán et al. 2024).

The prediction that unique environments would sustain more rare species (which would contribute less to β diversity, SCBD) was not supported by our results. While rare species are often associated with exclusive sites, a species' contribution to β diversity is not directly related to its rarity but rather to its occurrence variability across sampled sites (Legendre and De Cáceres 2013). Species found in few sites but with significant density or in highly distinct sites may exhibit high SCBD values. Conversely, very common but homogeneously distributed species tend to show low SCBD values (Louchart et al. 2024; Souza et al. 2025).

In our study, high‐density and widely distributed species like *Tintinnidium* sp. and *Limnostrombidium viride* had the highest SCBD, indicating that their density and spatial variation were key to their β diversity contribution (de Mello Cionek et al. 2022). Thus, SCBD reflects not just species presence/absence but their relative distribution and variability across environments, as discussed by Legendre and De Cáceres (2013).

Results showed that dam‐impacted environments (EID) had, on average, higher SCBD values than subsystems with lower direct dam exposure (ENID), contradicting the hypothesis that less affected areas would sustain rarer species with lower β diversity contributions. Although the ENID system has greater environmental heterogeneity (Souza‐Filho 2009), its SCBD values were generally lower, likely reflecting more similar species composition across habitats. This aligns with the SCBD definition, where widely distributed species with low spatial exclusivity contribute less to β diversity (Legendre and De Cáceres 2013).

Beyond spatial density variation, SCBD patterns may also be shaped by the combined effects of environmental and spatial filters. Factors like seasonality impose temporal changes in environmental conditions, acting stochastically and influencing ciliate community composition over time (Liu et al. 2025). Conversely, landscape spatial structure may serve as an additional filter, favouring species with similar functional traits or phylogenetic proximity, contributing to differentiated community organisation between systems (Oliveira et al. 2024).

Among the main factors structuring the ciliate community, both for ENID and EID, the environmental, temporal, and resource components stood out, with the environmental component explaining the largest portion of the data variation, regardless of the system's degree of impact. Studies have reported environmental filters as key regulators of aquatic communities (fish: Aleixo et al. 2025; zooplankton: Simões, Lansac‐Tôha, and Bonecker 2013), as they determine ecological processes such as nutrient cycling and productivity, in addition to affecting the availability and quality of food resources and, consequently, influencing the structuring of the entire food web (Leclerc et al. 2023). Thus, the importance of the resource component may be intrinsically linked to the significance of the environmental component, although the main resource groups related to the ciliate community differ between sites.

Regarding the temporal component, we expected it to play a stronger structuring role in the EID system due to the long‐term reduction in the frequency and intensity of natural flood pulses. However, our results showed that the temporal component was similarly important in both EID and ENID. This pattern reflects the hydrological linkage between the systems: although ENID is not directly regulated, the Ivinhema River receives downstream‐propagated effects of flow regulation from the Paraná River. As a result, changes in the timing and magnitude of floods in EID indirectly modify the flood regime in ENID as well. Consequently, disruptions to the natural flood pulses influence both systems simultaneously, reducing the expected differences between them.

Conservation units, such as the Ivinhema River Floodplain State Park, play a relevant role in maintaining environmental quality and preserving conditions closer to their natural pristine state, such as higher water turbidity levels and elevated nutrient concentrations (dos Santos Picapedra et al. 2025), thereby generating a positive impact on regional biodiversity and its ecosystem services. Numerous studies have demonstrated the effectiveness of these protected areas in recovering and conserving biodiversity across different biological groups (Silva et al. 2025; Urbano et al. 2025). However, the results of the present study suggest that even microbial communities with very short life cycles, such as planktonic ciliates, demonstrate the long‐term impact of the absence of flood pulses on their community organisation. In this context, the reduction in the intensity and frequency of floods appears to similarly impact the planktonic ciliate communities in the studied floodplain environments, both in the Paraná River, directly affected by dams (EID), and in the Ivinhema River (ENID), a tributary of the Paraná River with a significant conservation unit (Ivinhema State Park). Thus, considering the ecological relevance of aquatic microorganisms, including ciliates, for ecosystem functioning, the present study reinforces the idea that, in addition to preserving local environmental conditions ensured by conservation units, it is also essential for preserving the natural temporal dynamics of the flood pulse, as it acts as a key regulator of the structure and dynamics of these communities across all analysed environments.

Our inference about the role of damming should be interpreted in light of one limitation. Biological monitoring began in 2010, whereas the Porto Primavera Reservoir became operational in 1999. With this, the dataset therefore lacks a pre‐impoundment biological baseline and does not permit a strict before–after causal test. Our interpretation instead rests on two complementary lines of evidence: the comparative contrast between subsystems experiencing different degrees of direct hydrological regulation (EID vs. ENID), and the long‐term hydrological trend documented within the study period (Figure 2), namely the progressive decline in water level and in the frequency of connecting flood pulses above the 3.5 m threshold. This ongoing hydrological alteration is consistent with independent evidence of post‐impoundment change in the Paraná River flow regime, including reduced flood amplitude and attenuated pulses (Souza‐Filho 2009; Stevaux et al. 2009; Rocha 2010). Accordingly, we frame our findings as showing that dam‐mediated hydrological regulation is associated with divergent long‐term β diversity dynamics, rather than asserting that dam construction alone changes the planktonic ciliate community (Souza et al. 2026 in press).

Our results demonstrate that changes in the structure and composition of planktonic ciliate communities over time were strongly associated with dam‐related alterations, primarily through modifications of environmental factors previously regulated by natural processes. Protected areas, such as the Ivinhema River Floodplain State Park (ENID), exhibited relatively greater ecological stability, although still influenced by regional hydrological dynamics and temporal variability, whereas environments more directly influenced by dam regulation (EID) tended to show greater compositional fluctuations and gradual diversity loss. This contrast underscores the important role of conservation units in maintaining heterogeneous and functional habitats, thereby supporting the persistence of sensitive species.

However, the results also indicate that territorial protection alone is insufficient to ensure the ecological integrity of floodplains. The continuity of ecological processes in these systems depends on the maintenance of natural hydrological regimes, particularly flood pulses, which promote connectivity, organism dispersal, and community renewal. As previously demonstrated for fish communities, ciliates, small organisms with short life cycles, also reflect the long‐term effects of disrupted hydrological cycles, capturing both the influence of damming and the importance of natural pulses for diversity patterns and associated ecosystem services. Therefore, we recommend that reservoir management should incorporate water release strategies simulating natural flood pulses, promoting hydrological management that is more integrated with biodiversity conservation in regulated aquatic environments.

## Author Contributions

**Yasmin Rodrigues de Souza:** conceptualization, investigation, writing – original draft, methodology, validation, visualization, writing – review and editing, software, formal analysis, project administration, data curation, supervision. **Matheus Henrique de Oliveira de Matos:** conceptualization, investigation, methodology, validation, visualization, writing – review and editing, formal analysis, software, data curation. **Florencia M. Monti Areco:** conceptualization, investigation, formal analysis, data curation, writing – review and editing. **Melissa Progênio:** conceptualization, investigation, writing – review and editing. **Bianca Ramos de Meira:** conceptualization, investigation, writing – review and editing. **Andressa Crystine Souza‐Silva:** conceptualization, investigation, writing – review and editing. **Felipe Rafael de Oliveira:** conceptualization, investigation, writing – review and editing. **Luiz Felipe Machado Velho:** conceptualization, investigation, writing – original draft, methodology, validation, visualization, writing – review and editing, project administration, supervision, data curation.

## Funding

This work was supported by Conselho Nacional de Desenvolvimento Científico e Tecnológico; Coordenação de Aperfeiçoamento de Pessoal de Nível Superior; Centro de Pesquisa em Limnologia, Ictiologia e Aquicultura; Universidade Estadual de Maringá.

## Conflicts of Interest

The authors declare no conflicts of interest.

## Supporting information


**Data S1:** emi70421‐sup‐0001‐Supinfo.docx.
**Supporting Information: S1** Satellite image for the study area.
**Supporting Information: S2** Characteristics of the environments in each system.
**Table S1:** Minimum, maximum, and standard deviation values (in parentheses) of the environmental variables measured throughout the study period. The variables include air temperature, water temperature, dissolved oxygen in mg/L (DO mg) and in percentage (DO%), pH, conductivity, Secchi depth, turbidity, inorganic suspended solids (ISM), organic suspended solids (OSM), alkalinity, total nitrogen (TN), nitrate (NO_3_), ammonium (NH_4_), total phosphorus (TP), and phosphate (PO_4_).
**Table S2:** List of taxa recorded throughout the study period. The symbol (+) indicates the occurrence of the species in each sampled environment.
**Table S3:** SCBD values over the years in each system and environment.

## Data Availability

The data supporting the findings of this study are openly available in Zenodo at: https://doi.org/10.5281/zenodo.19613880. All data used in this study are available for review and will remain publicly accessible upon publication.
