# Supplementary material for: Long‐Term Changes in Planktonic Ciliate Beta Diversity Under Contrasting Hydrological Regulation in A River–Floodplain System
Source: Environ Microbiol. 2026 Sep 16;28(9):e70421. doi: 10.1111/1462-2920.70421 (PMC13579496; doi:10.1111/1462-2920.70421)
Supplement: Supplementary file 1 [file EMI-28-e70421-s001.docx]

**LONG-TERM CHANGES IN PLANKTONIC CILIATE BETA DIVERSITY UNDER CONTRASTING HYDROLOGICAL REGULATION IN A RIVER–FLOODPLAIN SYSTEM**

Yasmin Rodrigues de Souza¹*, Matheus Henrique de Oliveira de Matos¹, Florencia M. Monti Areco², Melissa Progênio¹, Bianca Ramos de Meira¹, Andressa Crystine Souza-Silva, Felipe Rafael de Oliveira^3^, Luiz Felipe Machado Velho¹

^1^ Universidade Estadual de Maringá, Programa de Pós-Graduação em Ecologia de Ambientes Aquáticos Continentais-PEA, Núcleo de Pesquisas em Limnologia, Ictiologia e Aquicultura, Av. Colombo, 5790, CEP: 87020-900, Campus Universitário - Maringá - PR, Brazil.

^2^ Centro de Ecología Aplicada del Litoral (CECOAL-CONICET). Ruta Provincial N.º 5, Corrientes, Argentina

^3^ Universidade do Estado do Mato Grosso (UNEMAT), Avenida Tancredo Neves, 1095, Cavalhada III, CEP 78217-900, Cáceres – MT, Brazil

***Correspondence author:** [yasmin.noris@gmail.com](mailto:yasmin.noris@gmail.com)

**ORCID:**

Yasmin Rodrigues de Souza:<https://orcid.org/0000-0002-5740-5442>

Matheus Henrique de Oliveira de Matos:<https://orcid.org/0000-0001-5984-2999>

Florencia M. Monti Areco <https://orcid.org/0000-0001-8234-4939>

Melissa Progênio https://orcid.org/0000-0002-0083-3358

Bianca Ramos de Meira<https://orcid.org/0000-0003-4961-038>

Andressa Crystine Souza-Silva https://orcid.org/0000-0002-4094-0465

Felipe Rafael de Oliveira https://orcid.org/0000-0002-7787-1440

Luiz Felipe Machado Velho<https://orcid.org/0000-0001-8111-4955>

**Supplementary material 1-** Satellite image for the study area.

**Supplementary material 2-** Characteristics of the environments in each system.

*Isolated shallow lakes*

***Ventura*** *shallow lake* ***– Ivinhema System***

Location: 22º51'23.7"S; 53º36'1.02"W. This elongated lake has an average depth of 2.16 meters, a length of 2,084.82 meters, a perimeter of 4,697.30 meters, and a surface area of 89.8 hectares. It is located 200 meters from the Ivinhema River, from which it is separated by a marginal levee 3 meters high. One of its banks is covered by forest (5%), while the opposite bank is dominated by grasses (95%).

***Osmar*** *shallow lake* ***– Paraná System***

Location: 22º46'26.64"S; 53º19'56.16"W. This is a temporary, isolated lake with an average depth of 1.1 meters and an approximate area of 0.006 hectares. It is located on Porto Rico Island and has an elongated shape, typical of the region’s fluvial islands. The substrate is sandstone and covered by pasture, with patches of riparian forest remnants classified as successional vegetation.

*Open shallow lakes*

***Patos*** *shallow lake* ***– Ivinhema System***

Location: 22º49'33.66"S; 53º33'9.9"W. Patos lake is the largest of the lakes sampled in this study. It has a glove-like shape, with inlets forming small bays, and an average depth of 3.50 meters. The lake covers an area of approximately 113.8 hectares, with a length of 2,065.6 meters and a perimeter of 14,783.8 meters. It is located just 10 meters from the Ivinhema River, separated by a levee only 0.5 meters high. The lake has a single connection to the river, measuring 8 meters in width.

***Garças*** *shallow lake* ***– Paraná System***

Location: 22º43'27.18"S; 53º13'4.56"W. Garças lake is located along the Paraná River. It has an average depth of 2.0 meters, a length of 2,128.1 meters, a surface area of 14.1 hectares, and a perimeter of 4,338.2 meters. Its banks are covered by grasses, and the surrounding area includes multiple strata of riparian vegetation.

***Ressaco do Pau Veio*** *shallow lake* ***– Paraná System***

Location: 22º44'50.76"S; 53º15'11.16"W. This waterbody connects to the right bank of the Paraná River. It has a length of 1,146.4 meters, a surface area of 3.0 hectares, and an average depth of 1.8 meters. Along its right bank, the vegetation includes ingá trees (*Inga uruguensis*), pasture fields, and Croton shrubs.

***Ipoitã Channel – Ivinhema System***

Location: 22º50'7.56"S; 53º33'43.5"W. The Ipoitã Channel is a sinuous watercourse connecting the Paraná River to the Ivinhema River. It has an average depth of 3.2 meters, and its banks are lined with shrub and tree vegetation, notably *Cecropia pachystachya* (embaúba).

*Rivers*

***Paraná River***

Location: 22º45'39.96"S; 53º15'7.44"W. This river section exhibits variable width, with the presence of islands and sandbars. In the sampled area, it has an average depth of 4.0 meters, although maximum depths may exceed 15 meters. The surrounding vegetation consists of open fields, with levees sparsely occupied by remnant trees of the original vegetation. The left bank (Paraná State) includes some settled areas, and some islands still support moderately disturbed arboreal vegetation.

***Ivinhema River***

Location: 22º47'59.64"S; 53º32'21.3"W. With an average depth of 3.9 meters, the Ivinhema River features diverse riparian vegetation along its banks, ranging from herbaceous cover to extensive areas of riparian forest at various stages of regeneration. In its lower course (sampled area), the river runs parallel to the Paraná River, connecting to the Baía River via the Curutuba Channel and to the Paraná River through the Ipoitã Channel and two additional channels.

**Table S1**. Minimum, maximum, and standard deviation values (in parentheses) of the environmental variables measured throughout the study period. The variables include air temperature, water temperature, dissolved oxygen in mg/L (DO mg) and in percentage (DO%), pH, conductivity, Secchi depth, turbidity, inorganic suspended solids (ISM), organic suspended solids (OSM), alkalinity, total nitrogen (TN), nitrate (NO_3_), ammonium (NH_4_), total phosphorus (TP), and phosphate (PO_4_).

|  | **EID** | **ENID** |
| --- | --- | --- |
| **Air temperature (°C)** | **16.5 - 32.5** | **9 - 36** |
|  | (3.8) | (4.4) |
| **Water temperature (°C)** | **16 - 32.8** | **17.7 - 33.3** |
|  | (3.6) | (3.4) |
| **Dissolved oxygen (mg/L)** | **0.34 - 11.7** | **0.18 - 13.15** |
|  | (2.5) | (2.12) |
| **Dissolved oxygen (%)** | **4.4 - 146.8** | **2.4 - 162.13** |
|  | (31) | (25.4) |
| **pH** | **4.35 - 8.46** | **5.46 - 9.49** |
|  | (0.63) | (0.54) |
| **Conductivity (µS/cm)** | **11.6 - 144.2** | **15.6 - 101.9** |
|  | (17) | (12.3) |
| **Secchi depth (m)** | **0 - 7.5** | **0.1 - 4.2** |
|  | (1.6) | (0.65) |
| **Turbidity (NTU)** | **0.07 - 51.92** | **0.36 - 147** |
|  | (7.9) | (25.56) |
| **ISM (µg/L)** | **0 - 3120** | **0 - 7880** |
|  | (428) | (829) |
| **OSM (µg/L)** | **0.06 - 2550** | **0.05 - 1520** |
|  | (405) | (199) |
| **Alkalinity (mg CaCO₃/L)** | **0.6227 - 660.2** | **3.78 - 576.1** |
|  | (196) | (144) |
| **Total nitrogen - TN (µg/L)** | **413 - 8014** | **100.7 - 3030** |
|  | (819) | (395) |
| **Nitrate (µg/L)** | **0 - 6757** | **0 - 1269.792** |
|  | (514) | (248) |
| **Ammonium (µg/L)** | **0 - 728** | **0 - 222** |
|  | (101) | (40) |
| **Total phosphorus - TP (µg/L)** | **0.062 - 435** | **0.14 - 530** |
|  | (70) | (44) |
| **Phosphate (µg/L)** | **0.09 - 139** | **0.09 - 113** |
|  | (22) | (16.8) |

**Table S2**. List of taxa recorded throughout the study period. The symbol (+) indicates the occurrence of the species in each sampled environment.

|  |  | **ENID** | | | | **EID** | | | |
| --- | --- | --- | --- | --- | --- | --- | --- | --- | --- |
| **order** | **taxon** | **Ipoitã Channel** | **Patos shallow lake** | **Ventura shallow lake** | **Ivinhema River** | **Garças shallow lake** | **Osmar shallow lake** | **Ressaco do Pau Veio shallow lake** | **Paraná River** |
| **Armophorida** | *Caenomorpha* spp Perty, 1852 |  | + |  | + |  |  |  |  |
| **Bursariomorphida** | *Bursaria truncatella* Müller, 1773 |  |  |  |  |  | + |  |  |
| **Bursariomorphida** | *Bursaridium pseudobursaria* (Faure-Fremiet, 1924) |  | + | + |  |  | + |  |  |
| **Chlamydodontida** | *Chilodonella uncinata* (Ehrenberg, 1838) | + | + | + | + | + | + | + |  |
| **Chlamydodontida** | *Pseudochilodonopsis algivora* (Kahl, 1931) |  |  | + |  | + |  |  |  |
| **Chlamydodontida** | *Trithigmostoma cucullulus* (Müller, 1786) |  |  |  |  |  |  | + |  |
| **Chlamydodontida** | *Trithigmostoma* sp. Jankowski, 1967 |  |  |  |  |  | + |  |  |
| **Choreotrichida** | *Codonella cratera* (Leidy, 1877) | + | + | + | + | + | + | + | + |
| **Choreotrichida** | *Tintinnidium fluviatile* (Stein, 1863) |  |  |  |  | + |  |  |  |
| **Choreotrichida** | *Tintinnidium* sp. Kent, 1881 | + | + | + | + | + | + | + | + |
| **Choreotrichida** | *Tintinnopsis lacustris* Faure-Fremiet, 1924 |  |  |  |  |  | + |  |  |
| **Choreotrichida** | *Tintinnopsis* sp. Stein, 1867 | + | + | + | + | + | + | + |  |
| **Colpodida** | *Colpoda cucculus* Müller, 1773 |  |  |  |  | + |  |  |  |
| **Colpodida** | *Colpoda inflata* Stokes, 1885 |  |  |  |  |  | + |  |  |
| **Colpodida** | *Colpoda magna* (Gruber, 1879) |  |  |  |  |  | + |  |  |
| **Colpodida** | *Colpoda* sp. Müller, 1773 |  | + | + | + | + | + | + |  |
| **Colpodida** | *Colpoda steinii* Maupas, 1883 |  |  |  |  | + |  |  |  |
| **Cyclotrichida** | *Cyclotrichium viride* Gajewskaja, 1933 |  |  |  |  | + |  |  |  |
| **Cyrtolophosidida** | *Cyrtolophosis mucicola* Stokes, 1885 | + | + | + | + |  | + | + | + |
| **Dileptida** | *Dileptus conspicuus* (Kahl, 1931) |  |  |  |  | + |  | + |  |
| **Dileptida** | *Dileptus margaritifer* (Ehrenberg, 1834) |  |  | + |  |  | + |  |  |
| **Dileptida** | *Dileptus* sp. Dujardin, 1841. |  |  |  |  |  | + |  |  |
| **Dileptida** | *Paradileptus elephantinus* (Svec, 1897) |  | + | + |  | + | + | + | + |
| **Dileptida** | *Paradileptus* sp. Wenrich, 1929 |  | + | + | + | + | + |  |  |
| **Dileptida** | *Trachelius ovum* (Ehrenberg, 1831) |  | + |  | + |  |  | + |  |
| **Endogenida** | *Tokophrya lemnarum* (Stein, 1859) |  |  |  |  |  |  |  | + |
| **Endogenida** | *Tokophrya* sp. Bütschli, 1889 | + |  |  |  |  |  |  |  |
| **Euplotida** | *Aspidisca cicada* (Müller, 1786) |  | + | + | + | + | + | + | + |
| **Euplotida** | *Aspidisca lynceus* (Müller, 1773) | + |  |  |  |  |  | + |  |
| **Euplotida** | *Euplotes aediculatus* Pierson, 1943 | + | + |  | + | + | + | + |  |
| **Euplotida** | *Euplotes affinis* Dujardin, 1842 | + | + | + |  |  |  |  |  |
| **Euplotida** | *Euplotes moebiusi* Kahl, 1932 |  |  |  | + | + | + |  |  |
| **Euplotida** | *Euplotes* sp. O.F. Müller, 1786 |  |  |  |  | + | + |  |  |
| **Exogenida** | *Sphaerophrya magna* Maupas, 1881 | + |  |  |  |  |  |  | + |
| **Gimnostomatida** | Gimnostomatida |  |  |  |  |  |  | + |  |
| **Gimnostomatida** | Gymno n id |  |  |  |  | + | + |  |  |
| **Haptorida** | *Didinium chlorelligerum* Kahl, 1935 |  | + |  |  | + |  |  |  |
| **Haptorida** | *Didinium nasutum* (Müller, 1773) | + | + |  |  | + | + | + | + |
| **Haptorida** | *Enchelyodon elegans* Kahl, 1926 | + |  | + |  |  | + | + |  |
| **Haptorida** | *Lacrymaria olor* (Müller, 1786) |  | + |  |  |  |  | + | + |
| **Haptorida** | *Monodinium balbiani* Fabre-Domergue, 1888 | + | + | + | + | + | + | + |  |
| **Haptorida** | *Monodinium* sp. Fabre-Domergue, 1888 |  |  | + |  | + |  |  |  |
| **Haptorida** | *Phialina* sp. Bory de St. Vincent, 1824 | + |  |  |  | + |  |  |  |
| **Haptorida** | *Teuthophrys trissulca trissulca* (Chatton & Beauchamp, 1923) |  |  | + |  |  |  |  |  |
| **Heterotrichida** | *Blepharisma* sp. Perty, 1849 |  |  | + |  |  | + |  |  |
| **Heterotrichida** | *Bursaria* sp. Müller, 1773. | + |  |  |  |  |  |  |  |
| **Heterotrichida** | *Climacostomum virens* (Ehrenberg, 1834) | + |  |  |  |  | + |  |  |
| **Heterotrichida** | *Linostomella vorticella* (Ehrenberg, 1834) |  | + | + |  | + | + |  |  |
| **Heterotrichida** | *Pseudoblepharisma tenue* Kahl, 1926 |  |  |  |  |  | + |  |  |
| **Heterotrichida** | *Spirostomum minus* Roux, 1901 |  | + | + |  | + |  |  |  |
| **Heterotrichida** | *Spirostomum teres* Claparède & Lachmann |  | + | + |  | + | + |  |  |
| **Heterotrichida** | *Stentor muelleri* Ehrenberg, 1832 |  |  | + |  |  |  |  |  |
| **Heterotrichida** | *Stentor multiformis* (Müller, 1786) Ehrenberg, 1838 |  | + | + |  | + |  |  |  |
| **Heterotrichida** | *Stentor niger* (Müller, 1773) | + | + | + |  | + |  | + |  |
| **Heterotrichida** | *Stentor roeselii* Ehrenberg, 1835 |  | + |  | + | + |  | + |  |
| **Heterotrichida** | *Stentor* sp. Oken, 1815 | + |  |  | + | + |  | + |  |
| **Hypotricha** | Hypotricha |  | + |  |  |  | + |  |  |
| **Hypotricha** | Hypotrichia n id |  | + |  | + |  |  |  |  |
| **Incertae sedis** | *Askenasia acrostomia* Krainer & Foissner, 1990 |  |  |  |  |  | + |  |  |
| **Incertae sedis** | *Askenasia volvox* (Eichwald, 1852) | + | + | + | + | + | + | + | + |
| **Incertae sedis** | Ciliado não identificado | + | + | + | + | + | + |  | + |
| **Incertae sedis** | *Mesodinium acarus* Stein, 1867 |  | + | + |  |  |  |  |  |
| **Incertae sedis** | *Mesodinium pulex* Claparède & Lachmann, 1858 | + | + | + | + | + | + | + | + |
| **Loxocephalida** | *Cinetochilum margaritaceum* (Ehrenberg, 1831) | + | + | + | + | + | + | + | + |
| **Loxocephalida** | *Dexiostricha granulosa* (Kent, 1881) | + | + |  |  | + |  |  |  |
| **Loxocephalida** | *Dexiotrichides centralis* (Stokes, 1885) | + |  |  |  | + | + |  |  |
| **Loxocephalida** | *Loxocephalus luridus* Eberhard, 1862 |  |  |  | + |  | + |  | + |
| **Loxodida** | *Loxodes magnum* Stokes, 188 | + |  | + | + |  |  | + |  |
| **Loxodida** | *Loxodes rostrum* (Müller, 1773) |  | + | + |  | + | + | + |  |
| **Metopida** | *Metopus* spp. sensu lato |  |  |  | + |  | + |  | + |
| **Microthoracida** | *Drepanomonas revoluta* Penard, 1922 |  | + | + |  | + |  |  |  |
| **Microthoracida** | *Hexotricha caudata* Lackey, 1925 |  |  |  |  |  |  | + |  |
| **Microthoracida** | *Microtorax pusilus* Engelmann, 1862 |  | + |  | + | + | + |  |  |
| **Microthoracida** | *Pseudomicrothorax agilis* Mermod, 1914 | + |  |  |  |  |  | + |  |
| **Nassulida** | *Nassula ornata* Ehrenberg, 1834 |  | + | + |  | + | + |  |  |
| **Nassulida** | *Nassula picta* (Greeff, 1888) |  | + |  |  |  |  | + |  |
| **Odontostomatida** | *Saprodinium* sp. Lauterborn, 1908 |  |  |  |  |  |  | + |  |
| **Oligotrichida** | *Limnostrombidium* sp. Krainer, 1932 | + |  | + |  | + | + | + | + |
| **Oligotrichida** | *Limnostrombidium viride* (Stein, 1867) | + | + | + | + | + | + | + |  |
| **Oligotrichida** | *Pelagostrombidium* sp. Krainer, 1991 |  | + |  |  | + |  | + |  |
| **Oligotrichida** | *Rimostrombidium humile* (Pénard, 1922) | + | + | + | + | + | + | + | + |
| **Oligotrichida** | *Rimostrombidium lacustris* (Foissner, Skogstad & Pratt, 1988) | + | + | + | + | + | + | + | + |
| **Oligotrichida** | *Rimostrombidium* sp. Jankowski, 1978 |  | + |  |  | + | + |  |  |
| **Oligotrichida** | *Rimostrombidium velox* Fauré-Fremiet, 1924) |  | + |  |  |  |  |  |  |
| **Ophryoglenida** | *Ophryoglena* sp. Ehrenberg, 1831 | + | + | + | + | + |  |  | + |
| **Peniculida** | *Disematostoma buetscheli* Lauterborn, 1894 |  |  | + |  |  | + |  |  |
| **Peniculida** | *Disematostoma tetraedricum* Kahl, 1931 | + | + | + | + | + | + | + |  |
| **Peniculida** | *Frontonia acuminata* Bütschli, 1889 |  | + |  | + | + | + | + |  |
| **Peniculida** | *Frontonia angusta* Kahl, 1931 |  |  |  | + |  | + |  |  |
| **Peniculida** | *Frontonia atra* Ehrenberg, 1833 | + | + | + | + | + | + | + | + |
| **Peniculida** | *Frontonia elliptica* Beardsley, 1902 |  |  |  |  | + | + |  |  |
| **Peniculida** | *Frontonia leucas* Ehrenberg, 1838 | + | + |  |  | + | + | + |  |
| **Peniculida** | *Frontonia* sp. Ehrenberg, 1838 |  |  | + |  |  | + |  |  |
| **Peniculida** | *Lembadion bullinum* (Müller, 1786) |  | + |  |  | + | + |  |  |
| **Peniculida** | *Lembadion lucens* (Maskell, 1887) | + | + | + | + | + | + | + | + |
| **Peniculida** | *Lembadion magnum* Kahl, 1931 | + | + |  |  |  | + | + |  |
| **Peniculida** | *Marituja pelagica* Gajewskaja, 1928 |  |  | + |  |  |  |  | + |
| **Peniculida** | *Paramecium aurelia* complex Ehrenberg, 1838 | + | + |  |  | + | + | + |  |
| **Peniculida** | *Paramecium bursaria* (Ehrenberg, 1831) | + | + |  |  | + | + | + |  |
| **Peniculida** | *Paramecium caudatum* Ehrenberg, 1838 | + | + | + | + | + | + | + | + |
| **Peniculida** | *Paramecium putrinum* Claparede & Lachmann, 1858 |  |  |  |  |  | + |  |  |
| **Peniculida** | *Paramecium* sp. O.F. Müller, 1773 |  |  |  |  |  |  | + |  |
| **Peniculida** | *Stokesia vernalis* Wenrich, 1929 | + | + | + | + | + | + | + |  |
| **Peritrichia** | Peritrichia |  |  |  |  | + |  |  |  |
| **Peritrichia** | Peritrichia n id | + | + | + | + | + | + | + |  |
| **Philasterida** | *Platynematum sociale* (Penard, 1922) Foissner, Berger & Kohmann, 1994 | + | + |  | + | + | + |  | + |
| **Philasterida** | *Uronema nigricans* (Müller, 1786) | + | + |  | + | + | + | + |  |
| **Philasterida** | *Urozona buetschli* Schewiakoff, 1889 |  |  | + |  |  |  |  |  |
| **Plagiopylida** | *Plagiopyla nasuta* Stein, 1860 |  | + |  |  |  |  |  |  |
| **Plagiopylida** | *Trimyema compressum* Lackey, 1925 |  |  |  | + |  |  |  |  |
| **Platyophryida** | *Platyophyra vorax* Kahl, 1926 | + | + |  | + | + | + | + | + |
| **Pleuronematida** | *Calyptotricha lanuginosa* (Penard, 1922) | + | + | + | + | + | + | + |  |
| **Pleuronematida** | *Ctedoctema acanthocryptum* Stokes, 1884 |  | + | + | + | + | + |  | + |
| **Pleuronematida** | *Cyclidium glaucoma* Müller, 1773 | + | + | + | + | + | + | + | + |
| **Pleuronematida** | *Cyclidium heptatricum* Schewiakoff, 1893 | + | + | + |  | + | + | + | + |
| **Pleuronematida** | *Philasterides armatus* (Kahl, 1926) | + | + | + | + | + | + | + | + |
| **Pleuronematida** | *Pleuronema coronatum* Kent, 1881 |  | + |  |  |  |  | + |  |
| **Pleurostomatida** | *Acineria incurvata* Dujardin, 1841 |  |  |  |  |  | + |  |  |
| **Pleurostomatida** | *Acineria uncinata* Tucolesco, 1962 | + | + | + |  | + |  | + | + |
| **Pleurostomatida** | *Amphileptus pleurosigma* (Stokes 1884) |  |  |  |  | + |  |  |  |
| **Pleurostomatida** | *Amphileptus* sp. Ehrenberg, 1830 |  |  |  |  | + |  |  |  |
| **Pleurostomatida** | *Litonotus alpestris* Foissner, 1978 |  | + |  | + |  | + | + |  |
| **Pleurostomatida** | *Litonotus cygnus* (Müller, 1773) |  |  |  |  |  | + |  |  |
| **Pleurostomatida** | *Litonotus fusidens* (Kahl, 1926) |  |  |  |  |  |  | + |  |
| **Pleurostomatida** | *Litonotus lamella* (Müller, 1773) | + | + |  |  | + | + |  |  |
| **Pleurostomatida** | *Litonotus varsaviensis* (Wrzesniowski, 1866) | + | + |  | + | + | + |  |  |
| **Pleurostomatida** | *Loxophyllum helus* (Stokes, 1884) Penard, 1922 |  | + |  |  |  |  |  |  |
| **Pleurostomatida** | Pleurostomatida | + |  |  |  |  |  |  |  |
| **Prorodontia** | *Coleps hirtus* (Müller, 1786) | + | + | + | + | + | + | + | + |
| **Prorodontia** | *Coleps hirtus hirtus* (Müller, 1786) | + | + | + |  |  |  |  |  |
| **Prorodontia** | *Coleps spetai* Foissner, 1984 | + | + | + | + | + | + | + | + |
| **Prorodontia** | *Holophrya discolor* Ehrenberg, 1833 | + | + | + | + | + | + | + | + |
| **Prorodontia** | *Holophrya ovum* Ehrenberg, 1831 | + | + | + | + | + | + | + |  |
| **Prorodontia** | *Holophrya* sp. Ehrenberg, 1831 |  | + |  |  |  | + | + |  |
| **Prorodontia** | *Holophrya teres* (Ehrenberg, 1833) | + | + |  | + | + | + | + |  |
| **Prorodontia** | *Lagynus elegans* (Engelmann,1862) |  |  |  |  |  | + |  |  |
| **Prorodontia** | *Pelagothrix chloreligera* Foissner, Berger & Schaumburg, 1999 | + | + | + |  |  |  |  |  |
| **Prorodontida** | *Balanion planctonicum* (Foissner, Oleksiv & Müller, 1990) | + | + | + | + | + | + | + | + |
| **Prorodontida** | *Bursellopsis spumosa* (Schmidt, 1920) | + | + | + |  |  | + | + |  |
| **Prorodontida** | *Placus luciae* (Kahl, 1926) | + | + |  | + |  | + | + |  |
| **Prorodontida** | *Plagiocampa rouxi* Kahl, 1926 | + |  |  | + |  |  |  |  |
| **Prorodontida** | *Prorodon ellipticus* (Kahl, 1930) |  | + |  |  | + |  | + |  |
| **Prorodontida** | *Urotricha farcta* Claparède & Lachmann, 1859 | + | + | + | + | + | + | + | + |
| **Prorodontida** | *Urotricha macrostoma* Foissner, 1983 |  |  |  |  | + |  |  |  |
| **Prorodontida** | *Urotricha pelagica* Kahl, 1935 |  |  | + |  |  |  |  |  |
| **Prorodontida** | *Urotricha* sp. Claparède & Lachmann, 1959 | + | + | + | + | + | + | + | + |
| **Prorodontida** | *Urotricha* sp1 |  |  | + |  |  |  |  |  |
| **Sessilida** | *Astylozoon fallax* Engelmann, 1862 |  |  | + |  |  |  |  |  |
| **Sessilida** | *Campanella umbellaria* (Linnaeus, 1758) | + | + | + | + |  | + |  |  |
| **Sessilida** | *Carchesium polypinum* (Linnaeus, 1758) |  | + | + |  | + | + | + |  |
| **Sessilida** | *Carchesium* sp. Ehrenberg, 1830 |  |  | + |  | + |  |  |  |
| **Sessilida** | *Epicarchesium pectinatum* (Zacharias, 1897) | + | + | + |  | + |  |  | + |
| **Sessilida** | *Epistylis anastatica* (Linnaeus, 1767) |  |  |  |  | + | + |  |  |
| **Sessilida** | *Epistylis coronata* Nusch, 1970 | + | + |  |  | + |  |  | + |
| **Sessilida** | *Epistylis galea* Ehrenberg, 1831 | + | + |  |  | + |  |  |  |
| **Sessilida** | *Epistylis hentscheli* Kahl, 1935 |  | + | + |  |  | + |  |  |
| **Sessilida** | *Epistylis nympharum* Engelmann, 1862 |  | + | + |  | + | + |  | + |
| **Sessilida** | *Epistylis procumbens* Ehrenberg, 1830 |  |  |  |  |  | + |  |  |
| **Sessilida** | *Epistylis pygmaeum* (Ehrenberg,1838) |  | + | + |  | + |  |  |  |
| **Sessilida** | Epistylis sp. Ehrenberg, 1830 | + | + | + |  | + | + |  |  |
| **Sessilida** | *Pelagovorticella natans* (Faure-Fremiet, 1924) | + | + |  |  | + |  |  |  |
| **Sessilida** | *Scyphidia rugosa* Dujardin, 1841 |  | + |  |  |  | + |  |  |
| **Sessilida** | *Thuricola* sp. Kent, 1881 | + |  |  | + |  |  |  |  |
| **Sessilida** | *Thuricola vasiformis* Hammann, 1952 | + |  |  |  |  |  |  |  |
| **Sessilida** | *Vaginicola* sp. | + |  |  |  | + |  |  |  |
| **Sessilida** | *Vorticella aquadulcis complex* Stokes, 1887 | + | + | + | + | + | + | + | + |
| **Sessilida** | *Vorticella campanula* Ehrenberg, 1831 | + | + | + | + | + | + | + | + |
| **Sessilida** | *Vorticella conochilli* Stokes, 1888 |  |  |  |  | + |  |  |  |
| **Sessilida** | *Vorticella convalaria* complex Linnaeus, 1758 | + | + | + | + | + | + | + | + |
| **Sessilida** | *Vorticella natans* Faure-Fremiet, 1924 |  |  |  |  | + |  |  |  |
| **Sessilida** | *Vorticella picta* (Ehrenberg, 1831) |  |  |  |  | + |  | + |  |
| **Sessilida** | Vorticella solta |  | + | + |  | + | + |  |  |
| **Sessilida** | *Vorticella* sp. Linnaeus, 1767 | + | + | + | + | + | + | + | + |
| **Spathidiid** | *Trachelophyllum apiculatum* (Perty, 1852) |  |  |  |  |  | + |  |  |
| **Spathidiid** | *Trachelophyllum* sp. Claparede & Lachmann, 1858 |  |  |  | + | + |  |  |  |
| **Spathidiida** | *Actinobolina radians* (Stein, 1867) |  |  |  |  | + | + | + |  |
| **Spathidiida** | *Actinobolina smalli* Holt, Lynn & Corliss, 1973 | + | + |  |  | + |  |  |  |
| **Spathidiida** | *Actinobolina* sp. Strand, 1928 |  |  | + |  |  |  |  | + |
| **Spathidiida** | *Actinobolina vorax* (Wenrich, 1929) |  | + |  | + | + | + | + |  |
| **Spathidiida** | *Enchelys gasterosteus* Kahl, 1926 |  | + | + |  | + | + | + |  |
| **Spathidiida** | *Enchelys* sp. Müller 1773 | + | + | + | + | + | + |  | + |
| **Spathidiida** | *Laginophrya acuminata* Kahl, 1935 | + | + | + | + | + | + | + |  |
| **Spathidiida** | *Laginophrya* sp. Stein, 1851 | + |  |  |  |  | + |  |  |
| **Spathidiida** | *Spathidium sensu lato* |  | + |  |  |  | + |  |  |
| **Stichotrichida** | *Halteria grandinella* (Müller, 1773) | + | + | + | + | + | + | + | + |
| **Stichotrichida** | *Halteria* sp. Dujardin, 1841 |  | + |  |  |  |  |  |  |
| **Stichotrichida** | *Holosticha pullaster* (Müller, 1773) |  | + |  |  |  |  |  |  |
| **Stichotrichida** | *Hypotrichidium conicum* Ilowaisky, 1921 |  |  |  |  | + | + |  |  |
| **Stichotrichida** | *Kerona pediculus* (Müller, 1773) Müller, 1786 |  |  |  |  | + |  |  |  |
| **Stichotrichida** | *Oxytricha chlorelligera* Kahl, 1932 |  | + |  | + | + | + |  |  |
| **Stichotrichida** | *Oxytricha ferruginea* Stein, 1859 |  |  |  |  |  | + |  |  |
| **Stichotrichida** | *Oxytricha hymenostoma* Stokes, 1887 | + |  |  |  |  |  |  |  |
| **Stichotrichida** | *Oxytricha setigera* Stokes, 1891 |  | + |  |  |  |  |  |  |
| **Stichotrichida** | *Oxytricha similis* Engelmann, 1862 |  | + |  |  | + |  |  |  |
| **Stichotrichida** | *Oxytricha* sp. Bory de St. Vincent, 1826 |  |  | + |  | + | + |  |  |
| **Stichotrichida** | *Paraurostyla* sp. Borror, 1972 |  |  |  |  |  | + |  |  |
| **Stichotrichida** | *Paraurostyla viridis* (Stein, 1859) |  | + |  |  |  | + |  |  |
| **Stichotrichida** | *Pelagohalteria* sp. Foissner, Skogstad & Pratt, 1988 |  |  |  |  |  | + |  |  |
| **Stichotrichida** | *Stichotricha aculeata* Wrzesniowski,1866 |  |  |  |  | + |  |  |  |
| **Stichotrichida** | *Stichotricha secunda* Perty, 1849 |  | + | + |  | + |  | + |  |
| **Stichotrichida** | *Stylonychia* sp. Ehrenberg, 1830 |  | + |  |  | + |  |  |  |
| **Stichotrichida** | *Stylonychia vorax* Stokes, 1885 |  |  |  |  |  | + | + |  |
| **Stichotrichida** | *Tetmemena pustulata* (Mueller, 1786) |  | + |  |  |  |  |  |  |
| **Stichotrichida** | *Uroleptus piscis* (Müller, 1773) |  |  |  |  |  | + |  |  |
| **Stichotrichida** | *Uroleptus* sp. Ehrenberg, 1831 |  |  |  |  | + |  |  |  |
| **Synhymeniida** | *Chilodontopsis depressa* (Perty, 1852) | + | + | + | + |  |  |  |  |
| **Synhymeniida** | *Nassulopsis elegans* (Ehrenberg, 1833) |  |  |  |  |  | + |  |  |
| **Tetrahymenida** | *Colpidium colpoda* (Losana, 1829) | + |  | + |  | + |  | + |  |
| **Tetrahymenida** | *Colpidium kleini* (Foissner, 1969) |  |  | + |  |  | + |  |  |
| **Tetrahymenida** | *Dexiostoma campylum* (Stokes, 1886) |  | + |  | + | + | + |  |  |
| **Tetrahymenida** | *Epenardia myriophylli* Corliss, 1971 |  |  | + |  |  | + |  |  |
| **Tetrahymenida** | *Glaucoma reniformis* Schewiakoff, 1893 | + | + |  | + | + | + | + |  |
| **Tetrahymenida** | *Glaucoma scintillans* Ehrenberg, 1830 |  |  |  |  |  | + | + |  |
| **Tetrahymenida** | *Tetrahymena pyriformis* (Ehrenberg, 1830) Furgason, 1940 | + | + | + | + | + | + | + | + |
| **Tetrahymenida** | *Tetrahymena* sp. |  |  | + |  |  |  |  |  |
| **Urocentrida** | *Urocentrum turbo* Müller, 1786 | + | + | + |  | + | + | + |  |
| **Urostylida** | *Urostyla grandis* Ehrenberg, 1830 |  |  | 1 |  |  |  |  |  |

**Table S3.** SCBD values over the years in each system and environment.

| **Year** | **Environment** | **Type** | **System** | **Species** | **Abundance (Ind L^-1^)** | **SCBD value** |
| --- | --- | --- | --- | --- | --- | --- |
| 2010 | Ivinhema river | River | ENID | *Tintinnidium* sp. | 3,500 | 0.011 |
| 2010 | Ventura lake | isolated Lake | ENID | *Tintinnidium* sp. | 49,500 | 0.09 |
| 2010 | Patos lake | Open Lake | ENID | *Rimnostrombidium humile* | 16,000 | 0.093 |
| 2010 | Ipoitã channel | Channel | ENID | *Tintinnidium* sp. | 1,500 | 0.00006 |
| 2010 | Paraná river | River | EID | *Tintinnidium* sp. | 80 | 0.094 |
| 2010 | Osmar lake | isolated Lake | EID | *Codonella cratera* | 140 | 0.174 |
| 2010 | Garças lake | Open Lake | EID | *Carchesium* sp. | 760 | 0.339 |
| 2010 | Pau véio lake | Channel | EID | *Urotricha farcta* | 280 | 0.166 |
| 2011 | Ivinhema river | River | ENID | *Vorticella aquadulcis* | 100 | 0.284 |
| 2011 | Ventura lake | isolated Lake | ENID | *Vorticella aquadulcis* | 1,975 | 0.432 |
| 2011 | Patos lake | Open Lake | ENID | *Urotricha farcta* | 225 | 0.176 |
| 2011 | Ipoitã channel | Channel | ENID | *Placus luciae* | 225 | 0.158 |
| 2011 | Paraná river | River | EID | *Tintinnidium* sp. | 320 | 0.142 |
| 2011 | Osmar lake | isolated Lake | EID | *Tintinnidium* sp. | 120 | 0.124 |
| 2011 | Garças lake | Open Lake | EID | *Tintinnidium* sp. | 2,820 | 0.313 |
| 2011 | Pau véio lake | Channel | EID | *Tintinnidium* sp. | 300 | 0.519 |
| 2012 | Ivinhema river | River | ENID | *Mesodinium pulex* | 100 | 0.35 |
| 2012 | Ventura lake | isolated Lake | ENID | *Vorticella aquadulcis* | 20,450 | 0.541 |
| 2012 | Patos lake | Open Lake | ENID | *Codonella cratera* | 800 | 0.013 |
| 2012 | Ipoitã channel | Channel | ENID | *Tintinnidium* sp. | 275 | 0.306 |
| 2012 | Paraná river | River | EID | *Tintinnidium* sp. | 280 | 0.0007 |
| 2012 | Osmar lake | isolated Lake | EID | *Rimnostrombidium lacustris* | 1,040 | 0.134 |
| 2012 | Garças lake | Open Lake | EID | *Carchesium polypinum* | 720 | 0.348 |
| 2012 | Pau véio lake | Channel | EID | *Tintinnidium* sp. | 500 | 0.497 |
| 2013 | Ivinhema river | River | ENID | *Limnostrombidium viride* | 150 | 0.63 |
| 2013 | Ventura lake | isolated Lake | ENID | *Balanion planctonicum* | 175 | 0.199 |
| 2013 | Patos lake | Open Lake | ENID | *Limnostrombidium viride* | 75 | 0.218 |
| 2013 | Ipoitã channel | Channel | ENID | *Rimnostrombidium humile* | 50 | 0.306 |
| 2013 | Paraná river | River | EID | *Actinobolina* sp. | 40 | 0.5 |
| 2013 | Osmar lake | isolated Lake | EID | *Codonella cratera* | 1,080 | 0.384 |
| 2013 | Garças lake | Open Lake | EID | *Codonella cratera* | 1,800 | 0.171 |
| 2013 | Pau véio lake | Channel | EID | *Epystilis galea* | 400 | 0.363 |
| 2014 | Ivinhema river | River | ENID | *Tintinnidium* sp. | 125 | 0.166 |
| 2014 | Ventura lake | isolated Lake | ENID | *Codonella cratera* | 425 | 0.216 |
| 2014 | Patos lake | Open Lake | ENID | *Vorticella aquadulcis* | 725 | 0.279 |
| 2014 | Ipoitã channel | Channel | ENID | *Tintinnidium* sp. | 2,775 | 0.47 |
| 2014 | Paraná river | River | EID | *Codonella cratera* | 100 | 0.235 |
| 2014 | Osmar lake | isolated Lake | EID | *Vorticella aquadulcis* | 2,960 | 0.305 |
| 2014 | Garças lake | Open Lake | EID | *Vorticella aquadulcis* | 500 | 0.118 |
| 2014 | Pau véio lake | Channel | EID | *Tintinnidium* sp. | 620 | 0.249 |
| 2015 | Ivinhema river | River | ENID | *Tintinnidium* sp. | 40 | 0.121 |
| 2015 | Ventura lake | isolated Lake | ENID | *Stokesia vernalis* | 325 | 0.166 |
| 2015 | Patos lake | Open Lake | ENID | *Coleps hirtus* | 1,200 | 0.151 |
| 2015 | Ipoitã channel | Channel | ENID | *Tintinnidium* sp. | 200 | 0.11 |
| 2015 | Paraná river | River | EID | *Vorticella aquadulcis* | 1,380 | 0.44 |
| 2015 | Osmar lake | isolated Lake | EID | *Epystilis nympharum* | 1,820 | 0.493 |
| 2015 | Garças lake | Open Lake | EID | *Halteria grandinella* | 200 | 0.006 |
| 2015 | Pau véio lake | Channel | EID | *Vorticella aquadulcis* | 380 | 0.257 |
| 2016 | Ivinhema river | River | ENID | *Balanion planctonicum* | 100 | 0.086 |
| 2016 | Ventura lake | isolated Lake | ENID | *Paramecium bursaria* | 675 | 0.109 |
| 2016 | Patos lake | Open Lake | ENID | *Tintinnidium* sp. | 2,400 | 0.033 |
| 2016 | Ipoitã channel | Channel | ENID | *Monodinium balbiani* | 1,125 | 0.177 |
| 2016 | Paraná river | River | EID | *Vorticella aquadulcis* | 240 | 0.215 |
| 2016 | Osmar lake | isolated Lake | EID | *Halteria grandinella* | 3,750 | 0.046 |
| 2016 | Garças lake | Open Lake | EID | *Coleps hirtus* | 380 | 0.213 |
| 2016 | Pau véio lake | Channel | EID | *Epicarchesium pectinatum* | 440 | 0.169 |
| 2017 | Ivinhema river | River | ENID | *Tintinnidium* sp. | 60 | 0.007 |
| 2017 | Ventura lake | isolated Lake | ENID | *Coleps hirtus* | 1,020 | 0.375 |
| 2017 | Patos lake | Open Lake | ENID | *Tintinnidium* sp. | 1,920 | 0.441 |
| 2017 | Ipoitã channel | Channel | ENID | *Halteria grandinella* | 80 | 0.112 |
| 2017 | Paraná river | River | EID | *Vorticella aquadulcis* | 40 | 0.246 |
| 2017 | Osmar lake | isolated Lake | EID | *Halteria grandinella* | 100 | 0.094 |
| 2017 | Garças lake | Open Lake | EID | *Tintinnidium* sp. | 75 | 0.353 |
| 2017 | Pau véio lake | Channel | EID | *Vorticella aquadulcis* | 60 | 0.127 |
| 2018 | Ivinhema river | River | ENID | *Cyclidium glaucoma* | 80 | 0.129 |
| 2018 | Ventura lake | isolated Lake | ENID | *Holophrya ovum* | 960 | 0.297 |
| 2018 | Patos lake | Open Lake | ENID | *Carchesium polypinum* | 2,240 | 0.262 |
| 2018 | Ipoitã channel | Channel | ENID | *Tintinnidium* sp. | 360 | 0.166 |
| 2018 | Paraná river | River | EID | *Tintinnidium* sp. | 80 | 0.37 |
| 2018 | Osmar lake | isolated Lake | EID | *Urotricha farcta* | 80 | 0.279 |
| 2018 | Garças lake | Open Lake | EID | *Codonella cratera* | 1,500 | 0.233 |
| 2018 | Pau véio lake | Channel | EID | *Coleps spetai* | 680 | 0.073 |
| 2019 | Ivinhema river | River | ENID | *Tintinnidium* sp. | 60 | 0.182 |
| 2019 | Ventura lake | isolated Lake | ENID | *Halteria grandinella* | 640 | 0.059 |
| 2019 | Patos lake | Open Lake | ENID | *Tintinnidium* sp. | 440 | 0.22 |
| 2019 | Ipoitã channel | Channel | ENID | *Rimnostrombidium lacustris* | 200 | 0.063 |
| 2019 | Paraná river | River | EID | *Epystilis nympharum* | 140 | 0.125 |
| 2019 | Osmar lake | isolated Lake | EID | *Tintinnidium* sp. | 500 | 0.237 |
| 2019 | Garças lake | Open Lake | EID | *Codonella cratera* | 280 | 0.167 |
| 2019 | Pau véio lake | Channel | EID | *Vorticella aquadulcis* | 80 | 0.134 |
| 2020 | Ivinhema river | River | ENID | *Vorticella aquadulcis* | 40 | 0.096 |
| 2020 | Ventura lake | isolated Lake | ENID | *Tintinnidium* sp. | 180 | 0.073 |
| 2020 | Patos lake | Open Lake | ENID | *Halteria grandinella* | 140 | 0.07 |
| 2020 | Ipoitã channel | Channel | ENID | *Vorticella aquadulcis* | 220 | 0.097 |
| 2020 | Paraná river | River | EID | *Cyclidium glaucoma* | 10 | 0.084 |
| 2020 | Osmar lake | isolated Lake | EID | *Vorticella aquadulcis* | 130 | 0.117 |
| 2020 | Garças lake | Open Lake | EID | *Coleps spetai* | 60 | 0.103 |
| 2020 | Pau véio lake | Channel | EID | *Tintinnidium* sp. | 10 | 0.077 |
| 2021 | Ivinhema river | River | ENID | *Urotricha farcta* | 60 | 0.179 |
| 2021 | Ventura lake | isolated Lake | ENID | *Coleps hirtus* | 60 | 0.149 |
| 2021 | Patos lake | Open Lake | ENID | *Coleps hirtus* | 80 | 0.15 |
| 2021 | Ipoitã channel | Channel | ENID | *Urotricha farcta* | 20 | 0.18 |
| 2021 | Paraná river | River | EID | *Tintinnidium* sp. | 20 | 0.084 |
| 2021 | Osmar lake | isolated Lake | EID | *Urotricha farcta* | 120 | 0.17 |
| 2021 | Garças lake | Open Lake | EID | *Urotricha* sp. | 20 | 0.038 |
| 2021 | Pau véio lake | Channel | EID | *Vorticella aquadulcis* | 160 | 0.101 |
| 2022 | Ivinhema river | River | ENID | *Tintinnidium* sp. | 100 | 0.375 |
| 2022 | Ventura lake | isolated Lake | ENID | *Codonella cratera* | 640 | 0.24 |
| 2022 | Patos lake | Open Lake | ENID | *Codonella cratera* | 1,920 | 0.181 |
| 2022 | Ipoitã channel | Channel | ENID | *Codonella cratera* | 220 | 0.186 |
| 2022 | Paraná river | River | EID | *Vorticella aquadulcis* | 120 | 0.172 |
| 2022 | Osmar lake | isolated Lake | EID | *Urotricha farcta* | 620 | 0.23 |
| 2022 | Garças lake | Open Lake | EID | *Codonella cratera* | 260 | 0.166 |
| 2022 | Pau véio lake | Channel | EID | *Urotricha farcta* | 150 | 0.094 |
| 2023 | Ivinhema river | River | ENID | *Vorticella aquadulcis* | 140 | 0.201 |
| 2023 | Ventura lake | isolated Lake | ENID | *Carchesium polypinum* | 1,920 | 0.294 |
| 2023 | Patos lake | Open Lake | ENID | *Carchesium polypinum* | 1,280 | 0.441 |
| 2023 | Ipoitã channel | Channel | ENID | *Rimnostrombidium humile* | 160 | 0.158 |
| 2023 | Paraná river | River | EID | *Stokesia vernalis* | 840 | 0.135 |
| 2023 | Osmar lake | isolated Lake | EID | *Halteria grandinella* | 110 | 0.098 |
| 2023 | Garças lake | Open Lake | EID | *Urotricha farcta* | 30 | 0.22 |
| 2023 | Pau véio lake | Channel | EID | *Vorticella aquadulcis* | 560 | 0.246 |
| 2024 | Ivinhema river | River | ENID | *Rimnostrombidium humile* | 100 | 0.153 |
| 2024 | Ventura lake | isolated Lake | ENID | *Coleps spetai* | 720 | 0.159 |
| 2024 | Patos lake | Open Lake | ENID | *Urotricha farcta* | 1,140 | 0.189 |
| 2024 | Ipoitã channel | Channel | ENID | *Urotricha farcta* | 120 | 0.071 |
| 2024 | Paraná river | River | EID | *Didinium nasutum* | 587 | 0.179 |
| 2024 | Osmar lake | isolated Lake | EID | *Vorticella aquadulcis* | 120 | 0.2 |
| 2024 | Garças lake | Open Lake | EID | *Cyclidium glaucoma* | 70 | 0.139 |
| 2024 | Pau véio lake | Channel | EID | *Vorticella aquadulcis* | 80 | 0.211 |
